# Modular and Molecular Optimization of a LOV (Light–Oxygen–Voltage)-Based Optogenetic Switch in Yeast

**DOI:** 10.3390/ijms22168538

**Published:** 2021-08-09

**Authors:** Andrés Romero, Vicente Rojas, Verónica Delgado, Francisco Salinas, Luis F. Larrondo

**Affiliations:** 1 Departamento de Genética Molecular y Microbiología, Facultad de Ciencias Biológicas, Pontificia Universidad Católica de Chile, Santiago 8331150, Chile; andres.romero.q@ug.uchile.cl (A.R.); vlrojas@uc.cl (V.R.); vmdelgado@uc.cl (V.D.); 2 ANID–Millennium Science Initiative–Millennium Institute for Integrative Biology (iBIO), Santiago 8331150, Chile; francisco.salinas@uach.cl; 3 Instituto de Bioquímica y Microbiología, Facultad de Ciencias, Universidad Austral de Chile, Valdivia 5090000, Chile

**Keywords:** yeast, optogenetics, blue-light switch, gene expression

## Abstract

Optogenetic switches allow light-controlled gene expression with reversible and spatiotemporal resolution. In *Saccharomyces cerevisiae*, optogenetic tools hold great potential for a variety of metabolic engineering and biotechnology applications. In this work, we report on the modular optimization of the fungal light–oxygen–voltage (FUN-LOV) system, an optogenetic switch based on photoreceptors from the fungus *Neurospora crassa*. We also describe new switch variants obtained by replacing the Gal4 DNA-binding domain (DBD) of FUN-LOV with nine different DBDs from yeast transcription factors of the zinc cluster family. Among the tested modules, the variant carrying the Hap1p DBD, which we call “HAP-LOV”, displayed higher levels of luciferase expression upon induction compared to FUN-LOV. Further, the combination of the Hap1p DBD with either p65 or VP16 activation domains also resulted in higher levels of reporter expression compared to the original switch. Finally, we assessed the effects of the plasmid copy number and promoter strength controlling the expression of the FUN-LOV and HAP-LOV components, and observed that when low-copy plasmids and strong promoters were used, a stronger response was achieved in both systems. Altogether, we describe a new set of blue-light optogenetic switches carrying different protein modules, which expands the available suite of optogenetic tools in yeast and can additionally be applied to other systems.

## 1. Introduction

Optogenetics is an approach that began to gain attention when targeted light-activated neurotransmission, utilizing channelrhodopsin-2, was first described [1]. Since then, new optogenetic tools have been rapidly implemented in multiple biological platforms, enabling light control not only of ion currents, but of a vast set of biological processes [2,3]. In recent years, the eukaryotic model organism *Saccharomyces cerevisiae* (budding yeast) has become a salient chassis for optogenetics [4]. In this microorganism, numerous optogenetic devices have been developed and used to control different cellular processes by light, including protein activity reconstitution, subcellular protein localization, protein degradation, and tunable transcription [4,5,6,7].

Different blue-light optogenetic systems have been implemented in yeast to control gene expression, with remarkable applications in metabolic engineering and yeast biotechnology [8,9,10,11,12]. Among them, the OptoEXP and OptoINVRT optogenetic systems, both based on the blue-light photoreceptor EL222 from *Erythrobacter litoralis*, have been used to redirect the glycolytic metabolic flux depending on the applied light or dark regimens [10,11,12]. Similarly, the yLightOn optogenetic switch is a single component system based on the light–oxygen–voltage (LOV) domain of the protein VIVID (VVD), a blue-light photoreceptor from the filamentous fungus *Neurospora crassa* [9]. This system can be used to control gene expression and protein stability and has allowed, for instance, fine tuning of the yeast cell cycle upon light stimulation [9]. Another LOV-containing protein in *N. crassa* is White Collar-1 (WC-1), a blue-light photoreceptor and transcription factor (TF) that interacts with WC-2, forming the White Collar complex (WCC), which is the core component of the Neurospora circadian clock and is responsible for the transcriptional activation of numerous genes in response to light [13,14]. Among the latter is *vvd* and, as VVD is produced, it interacts with WC-1 through a LOV–LOV interaction, attenuating the transcriptional activity of the WCC in a process known as photoadaptation [15]. The LOV–LOV interaction of WC-1 and VVD was exploited for the development of the optogenetic switch named fungal light–oxygen–voltage (FUN-LOV), which allows accurate light-controlled transcription, with a wide dynamic range and low background expression [8]. FUN-LOV capabilities have been tested both in the context of heterologous protein production and easily scorable yeast phenotypes, such as flocculation [8]. Overall, these examples highlight the tremendous potential of optogenetics in the control of different yeast cellular processes.

The FUN-LOV switch is based on a yeast two-hybrid (Y2H) architecture [16], where the Gal4 DNA-binding domain (DBD) and activation domain (AD) are connected to the WC-1 and VVD LOV domains, respectively [8]. Thus, upon blue light stimulation, the LOV–LOV interaction of WC-1 and VVD enables the reconstitution of a functional TF that activates the expression of a target gene placed under the control of the cognate *GAL1* promoter (*P_GAL1_*) [8]. The components of the FUN-LOV system, on the other hand, are under the control of the *ADH1* promoter (*P_ADH1_*) and are encoded in two separate multicopy plasmids [8]. This may generate differences in the expression levels of the target gene due to plasmid copy number variations or the *P_ADH1_* strength. Recently, the molecular optimization of a blue-light optogenetic switch based on the interaction of CRY2 (cryptochrome 2) and CIB1 proteins was reported [17]. This optogenetic switch is encoded by a low copy number plasmid and under the control of a promoter with intermediate strength, resulting in distinctive expression levels of a CRY2–CIB1 target gene [17].

Gal4p has a well-known modular nature, where its DBD and AD domains can function as separate and independent modules [18,19]. For instance, the replacement of the Gal4 DBD by the LexA DBD from *Escherichia coli* generates a functional TF that recognizes the LexA operator sequence [18]. Similarly, the AD of Gal4p has been replaced by different ADs, such as B42 [20] from *E. coli* or VP16 from Herpes virus [21], generating functional TFs that are capable of recruiting and activating the transcriptional machinery. Gal4p belongs to the Zn(II)_2_Cyc_6_ family of TFs, a fungal zinc cluster family of transcriptional regulators involved in different biological processes in yeast, such as carbon and nitrogen metabolism, chromatin remodeling, cell cycle, stress responses, and respiration [22,23]. This family of modular TFs is composed of more than 46 proteins in yeast [22], whose DBDs could, in theory, be used to replace the Gal4 DBD utilized in FUN-LOV, thereby changing promoter specificity and providing users with another level of control over the expression levels of the target genes. Altogether, the modular feature of this class of TFs suggests that the DBD and AD of the FUN-LOV switch could be replaced with different building blocks, potentially yielding optogenetic systems capable of distinct levels of gene expression. This could expand the repertoire of orthogonal switches available to other organisms, particularly animal models, where Gal4-based systems have already proven extremely useful [24,25,26,27].

In this work, we performed a modular and molecular optimization of the FUN-LOV optogenetic switch in yeast, assessing the characteristics of different variants. We replaced the Gal4 DBD and AD of FUN-LOV with different modules of similar functionality and observed that the use of the Hap1p DBD (in what we called the “HAP-LOV” system) yielded increased expression of the reporter (luciferase) compared to FUN-LOV upon blue-light stimulation. We then evaluated the impact of both plasmid copy number and the strength of the promoter controlling the expression of FUN-LOV and HAP-LOV components, observing a strong increase in luciferase levels upon stimulation when FUN-LOV or HAP-LOV parts were encoded in low copy number plasmids and placed under the control of a strong promoter, such as *TDH3*. Altogether, we describe a new set of blue-light optogenetic switches in yeast, carrying different modules that can be exchanged to achieve different ratios of gene expression, which not only expands the available suite of yeast optogenetic modules, but also that of potential orthogonal controllers for animal models.

## 2. Results and Discussion

### 2.1. Modular Optimization of the FUN-LOV Optogenetic Switch

**Figure 1:**
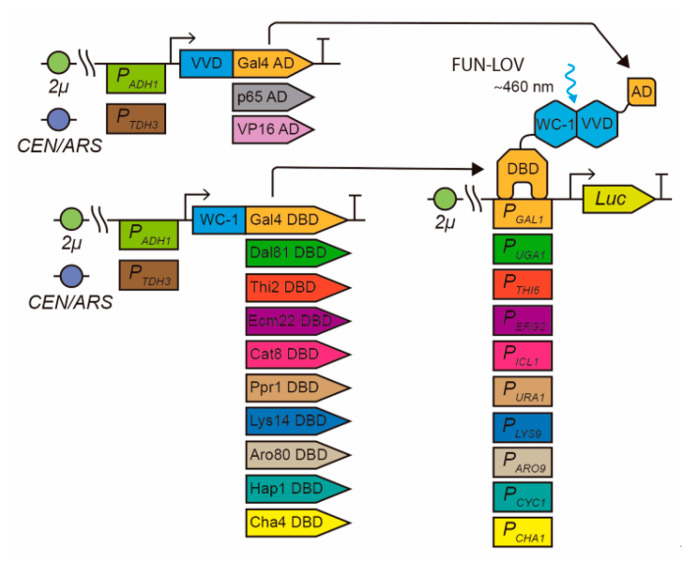
Modular and molecular optimization strategy used in the FUN-LOV optogenetic switch. For the modular optimization of FUN-LOV, the Gal4p DNA-binding domain (DBD) module was replaced with nine different DBDs belonging to the Zinc cluster family of transcription factors. The Gal4p activation domain (AD) module was replaced with the p65 and VP16 ADs. For the molecular optimization of FUN-LOV, the components were cloned into multicopy plasmids (2µ replication origin) or low-copy plasmids (CEN/ARS) and expressed under the control of promoters of different strengths (*P_ADH1_* or *P_TDH3_*).

Initially, we selected nine different TFs from the zinc cluster family (Zn(II)_2_Cys_6_) that have previously described DBDs and known target promoters (Table 1). We confirmed the N-terminal position of the DBDs in the amino acid (aa) sequence of each selected TF using Pfam [28], and chose a region of 150 aa containing the DBDs (Table 1). We used the DBDs from these nine TFs to replace the DBD module of the FUN-LOV switch, testing them by checking luciferase expression under the control of selected cognate promoters (Figure 1). We evaluated the yeast strains carrying the nine different variants of FUN-LOV using a single 120 min blue-light pulse (Supplementary Figure S1), which was administered during the mid-exponential phase (Supplementary Figure S2). Among the assayed strains, the one carrying the Hap1p DBD, which recognizes the *CYC1* promoter (*P_CYC1_*) [29], displayed a clear increase in luciferase activity (a good proxy of expression levels) in response to the blue-light pulse compared to the control, and this was rapidly reversed in the absence of the stimulus (Supplementary Figure S1). Hap1p is a TF that regulates gene expression in response to heme and oxygen levels [30].

**Figure 2:**
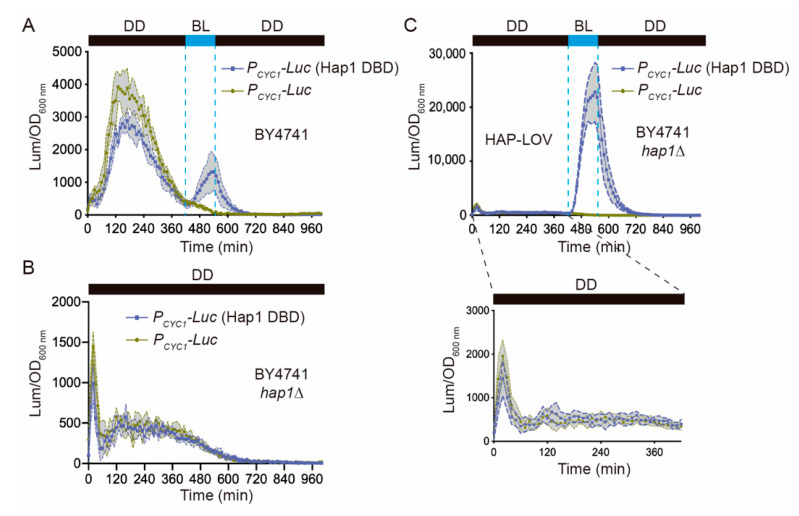
The HAP-LOV switch responds to light and its activity is higher in the absence of the endogenous Hap1p. Luciferase activity in response to HAP-LOV in the BY4741 or BY4741 *hap1∆* strains harboring the *CYC1* (*P_CYC1_*) promoter controlling luciferase (*Luc*) expression: (**A**) changes in reporter levels upon a blue-light (BL) pulse of 120 min (dashed lines) in the BY4741 strain; (**B**) background luciferase expression under constant darkness condition (DD) in the BY4741 *hap1∆* strain; (**C**) BL pulse of 120 min (dashed lines) in the BY4741 *hap1∆* strain. The background luciferase expression prior to the BL pulse has been magnified. In all panels, the yeast strain carrying only the *CYC1* (*P_CYC1_*) promoter controlling luciferase (*Luc*) expression, but not carrying HAP-LOV, is shown as a control. The graphs show the average of normalized luciferase expression in six biological replicates, with the standard deviation represented as a shaded region.

Notably, the Hap1p DBD system yielded strong background levels of luciferase expression even in the dark, in the absence of any light stimulus (Figure 2A). We hypothesized that this was due to the presence of the endogenous Hap1p, which resulted in basal CYC1 promoter activity. To address this, we deleted the *HAP1* gene (*hap1∆*) in the BY4741 strain and repeated the experiments, confirming a strong reduction of the luciferase background expression in darkness (Figure 2B). Notably, the BY4741 *hap1∆* strain showed a strong increase in luciferase expression when it was stimulated with a blue-light pulse of 120 min, which we surmised was due to the lack of competition between the endogenous and the recombinant Hap1 proteins (Figure 2C). The effect of the endogenous TF on the background expression of the new optogenetic system was also confirmed by analyzing the FUN-LOV variant carrying the Cha4p DBD (Supplementary Figure S1, panel I). In this system, termed CHA-LOV, deletion of the *CHA4* gene (*cha4∆*), encoding the endogenous Cha4 TF, also resulted in a strong reduction of luciferase background levels in darkness (Supplementary Figure S3). In addition, an increase in luciferase expression was observed upon a single blue-light pulse stimulation or when using different illumination regimens (Supplementary Figure S3). This effect was also previously observed in the original FUN-LOV switch, where deletion of the genes encoding the Gal4 TF (*gal4∆*) and its repressor Gal80p (*gal80∆*) diminished luciferase background expression in darkness while enhancing light responses [8]. Therefore, for optogenetic switches carrying DBDs from endogenous TFs, deletion of the corresponding TF encoding *loci* notably reduces background activation and increases light-induction responses.

**Figure 3:**
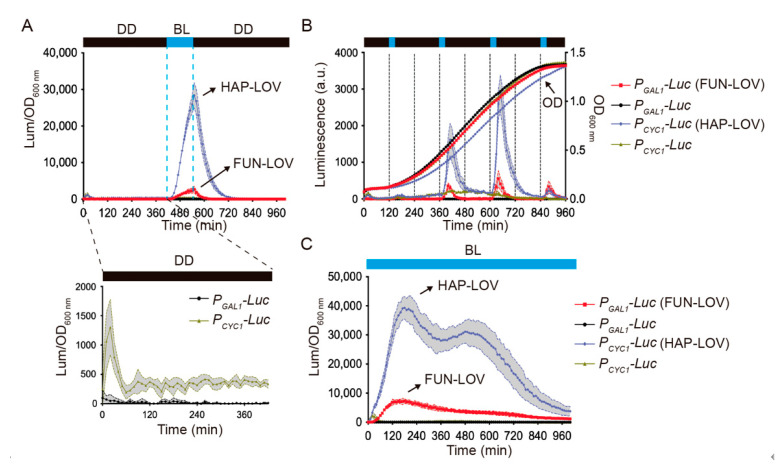
HAP-LOV provides strong and dynamic control of target gene expression. The graphs present changes in the luciferase reporter activity achieved by the FUN-LOV and HAP-LOV optogenetic switches under different illumination regimens. (**A**) Blue-light (BL) pulse of 120 min (dashed lines) for the yeast strains carrying the FUN-LOV and HAP-LOV switches, as indicated. The background luciferase expression prior to the BL pulse has been magnified. The yeast strains carrying the FUN-LOV and HAP-LOV switches were subjected to BL pulses of 30 min every 4 h (**B**) or constant BL illumination (**C**). In panel B, the raw luciferase expression and optical density (OD) at 600 nm is shown for yeast strains carrying the FUN-LOV and HAP-LOV switches. In all panels, the yeast strains carrying the *GAL1* (*P_GAL1_*) or *CYC1* (*P_CYC1_*) promoters controlling luciferase (*Luc*) expression, but lacking the corresponding switches, were used as controls. The graphs show the average of raw or normalized luciferase expression in six biological replicates, with the standard deviation represented as a shaded region. Abbreviations: DD, constant darkness; a.u., arbitrary units of luminescence.

This work focuses on characterizing the FUN-LOV variant carrying the Hap1p DBD, which was termed HAP-LOV (Figure 2C). In order to compare the luciferase expression reached by HAP-LOV to that of the FUN-LOV switch, we assayed the response of each system under different illumination regimes: a single 120 min blue-light pulse, 30 or 60 min pulses every 4 h, cycles of 120 min of blue-light followed by 120 min of darkness, or constant blue-light illumination (Figure 3 and Supplementary Figure S4). In all cases, the HAP-LOV switch displayed a higher background luciferase expression in darkness compared to FUN-LOV (Figure 3A). However, HAP-LOV showed higher luciferase levels compared to FUN-LOV in all the assayed illumination regimens (Figure 3B,C and Supplementary Figures S4 and S5). Interestingly, using blue-light pulses of 30 min every 4 h (Figure 3B), the maximal peak of luciferase expression for FUN-LOV and HAP-LOV coincided with the exponential growth phase of the yeast culture (Figure 3B). Furthermore, an increment in the blue-light pulse duration from 30 min to 2 h augmented the luciferase expression in FUN-LOV and HAP-LOV switches (Supplementary Figure S4). These results confirm previously described observations for the FUN-LOV switch, namely that the maximal luciferase expression was reached in the exponential growth phase and the length of the blue-light pulse resulted in increased expression of the reporter [8]. Altogether, the replacement of the Gal4p DBD by that of Hap1p in the FUN-LOV optogenetic switch yields overall higher basal reporter expression in darkness (*hap1∆* strain), but also leads to a stronger increase in luciferase levels upon blue-light stimuli (Figure 3), confirming the FUN-LOV modularity at the DBD level.

**Figure 4:**
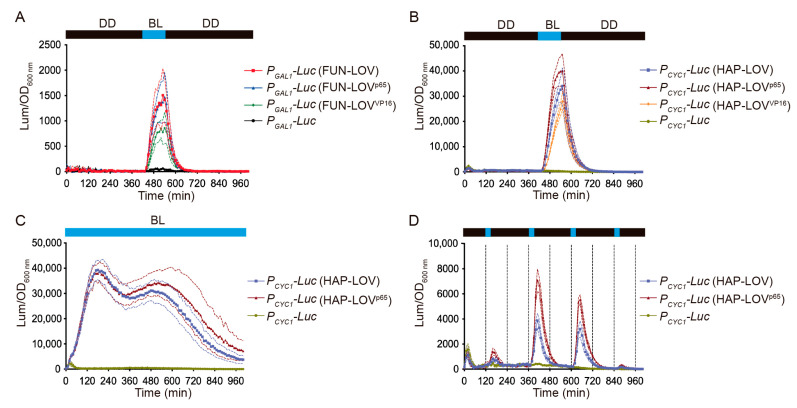
Normalized luciferase expression in the context of FUN-LOV and HAP-LOV systems carrying different activation domains (ADs). Yeast strains carrying the FUN-LOV (**A**) or HAP-LOV (**B**) systems and containing different ADs (VP16 or p65) were subjected to a blue-light (BL) pulse of 120 min. Similarly, yeast strains carrying the HAP-LOV and HAP-LOV^p65^ systems were subject to constant BL (**C**) and BL pulses of 30 min every 4 h (**D**). In all panels, the yeast strains carrying the *GAL1* (*P_GAL1_*) or *CYC1* (*P_CYC1_*) promoters controlling luciferase (*Luc*) expression, but lacking the corresponding switches, were used as controls. The graphs show the average of normalized luciferase expression in six biological replicates, with the standard deviation represented as regions between dashed lines.

We then evaluated the modularity of FUN-LOV at the AD level, replacing the Gal4 AD with the VP16 and p65 ADs (Figure 4A). We did not observe statistically significant differences (one-way ANOVA and Tukey’s multiple comparisons test, *p* > 0.99) in luciferase expression between the FUN-LOV system and its variants carrying VP16 or p65 upon blue-light illumination (Figure 4A and Supplementary Table S1). Similarly, we replaced the Gal4 AD of HAP-LOV with VP16 and p65 ADs. In this switch, on the other hand, we did observe a statistically significant increase in luciferase levels in the HAP-LOV version carrying the p65 AD (HAP-LOV^p65^) upon blue-light stimulation (Figure 4B,C and Supplementary Table S1). The differences in luciferase expression attained by HAP-LOV and HAP-LOV^p65^ were evident when we used blue-light pulses of different durations (Figure 4D and Supplementary Figure S6). Altogether, the replacement of Gal4 AD by VP16 or p65 ADs in the original FUN-LOV optogenetic switch did not have an important effect in expression levels upon blue-light stimulation. However, the HAP-LOV switch exhibited different behavior, where the replacement of the Gal4 AD by p65 increased the luciferase expression in response to blue light. Therefore, the FUN-LOV switch is modular at the DBD and AD levels, where the replacement of the DBD module (by Hap1p DBD) results in an increased response of the target gene, likely due to the transcriptional landscape of the cognate promoter.

### 2.2. Molecular Optimization of the FUN-LOV and HAP-LOV Switches

**Figure 5:**
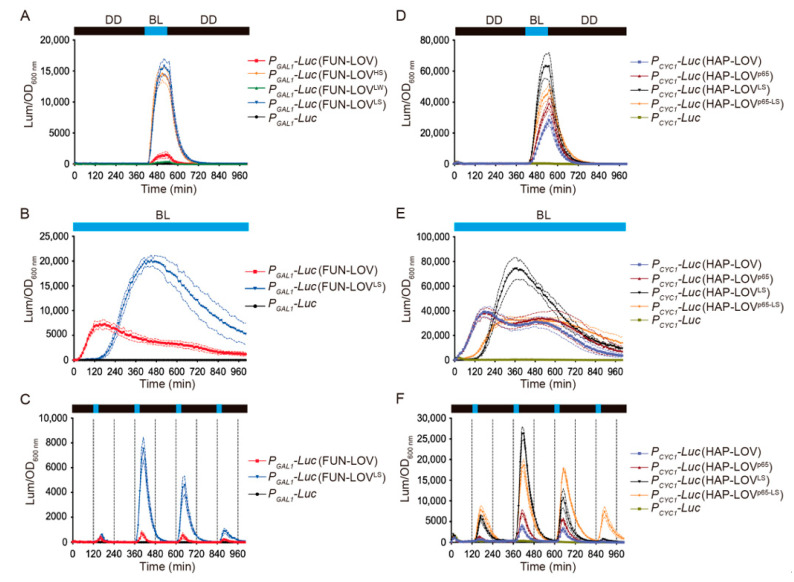
Molecular optimization of the promoter strength and plasmid copy number for the FUN-LOV and HAP-LOV optogenetic systems. (**A**) Yeast strains containing the FUN-LOV switch, or its variants, were subjected to a blue-light (BL) pulse of 120 min (dashed lines), and two of them (FUN-LOV and FUN-LOV^LS^) were subjected to constant BL illumination (**B**) or BL pulses of 30 min every 4 h (**C**). (**D**) Yeast strains carrying the HAP-LOV switch, or its variants, were subjected to a BL pulse of 120 min (dashed lines), constant BL illumination (**E**), or BL pulses of 30 min every 4 h (**F**). In all panels, yeast strains carrying the *GAL1* (*P_GAL1_*) or *CYC1* (*P_CYC1_*) promoters controlling luciferase (*Luc*) expression, but lacking the corresponding switches, were used as controls. The graphs show the average of normalized luciferase expression in six biological replicates, with standard deviation represented as regions between dashed lines. Abbreviations: H, high copy plasmid; L, low copy plasmid; S, strong promoter; W, weak promoter.

The copy number of the plasmid in which an optogenetic switch is encoded and the strength of the promoter controlling expression of the components of the optogenetic switch are key factors impacting the light response of the target gene [17]. In this sense, the FUN-LOV and HAP-LOV switches are encoded in multicopy plasmids of the pRS420 series [47], and their components are expressed under the control of *P_ADH1_*, which is a rather weak constitutive yeast promoter [48]. Thereby, we evaluated the behavior of FUN-LOV and HAP-LOV when their components are encoded in low-copy plasmids (pRS300 series) [49] and placed under the control of a strong constitutive promoter, such as *TDH3* (*P_TDH3_*) [48]. Initially, we assayed the FUN-LOV switch using different combinations: high (H)-copy plasmids (pRS420 series) and the strong (S) *TDH3* promoter (FUN-LOV^HS^), low (L)-copy plasmids (pRS300 series) and the weak (W) *ADH1* promoter (FUN-LOV^LW^), and low (L)-copy plasmids (pRS300 series) and the strong (S) *TDH3* promoter (FUN-LOV^LS^). Among these versions, the FUN-LOV^HS^ (HS: high copy, strong promoter) and FUN-LOV^LS^ (LS: low copy, strong promoter) displayed strong increases in luciferase expression upon blue-light induction (Figure 5A). Importantly, the yeast strain carrying FUN-LOV^HS^ exhibited reduced growth kinetics (Supplementary Figure S7), suggesting a possible metabolic burden due to the combination of FUN-LOV expression from multicopy plasmids with the use of a strong promoter [50]. Thus, we further characterized the FUN-LOV^LS^ system using different illumination regimes, including constant blue light and blue-light pulses of variable duration (Figure 5B,C; Supplementary Figure S7). In all the assayed illumination regimes, FUN-LOV^LS^ exhibited a strong increase in luciferase levels compared to the original FUN-LOV switch (Figure 5A–C). Altogether, molecular optimization of FUN-LOV consisting of encoding the components in low-copy plasmids and placing them under the control of a strong promoter greatly increased the level of target gene expression achieved upon blue-light stimulation.

In a similar way, we evaluated HAP-LOV and HAP-LOV^p65^ switches using low (L)-copy plasmids (pRS300 series) and the strong (S) *TDH3* promoter, resulting in the HAP-LOV^LS^ and HAP-LOV^p65-LS^ versions, respectively. Interestingly, we observed a progressive increase in luciferase levels when cultures were illuminated with a 120 min blue-light pulse as follows: HAP-LOV^LS^ > HAP-LOV^p65-LS^ > HAP-LOV^p65^ > HAP-LOV (Figure 5D). We further characterized these systems using different illumination regimes, including constant blue light and blue-light pulses of variable duration (Figure 5E,F Supplementary Figure S8). In all assayed illumination conditions, HAP-LOV^LS^ showed the highest increase in reporter expression (Figure 5E,F; Supplementary Figure S8). Altogether, molecular optimization of the HAP-LOV and HAP-LOV^p65^ switches consisting of encoding the components in low-copy plasmids and commanding their expression with a strong promoter resulted in a marked increase in the levels of target gene expression upon blue-light stimulation.

### 2.3. Comparing Different Optogenetic Systems

**Figure 6:**
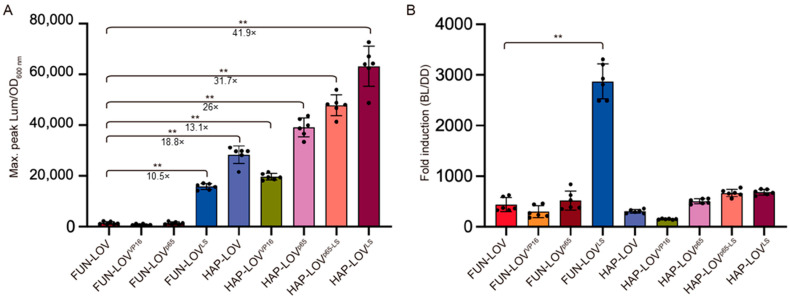
Dynamic range exhibited by the different optogenetic systems tested in this work. (**A**) The maximal peak of normalized luciferase expression for each optogenetic system was measured during a blue-light pulse of 120 min. (**B**) Luciferase activity fold-induction for each optogenetic system upon a blue-light (BL) pulse of 120 min, compared to the constant darkness (DD) condition. The graph shows the average of six biological replicas. ** Double asterisks indicate a statistically significant difference with respect to the original FUN-LOV system (*t*-test, *p* < 0.01).

In order to compare the different versions of the FUN-LOV and HAP-LOV optogenetic switches, we selected the maximal level of luciferase expression reached by each system after a discrete 120 min blue-light pulse. By using this approach, we observed that FUN-LOV^LS^ yielded a 10.5-fold increase in luciferase expression compared to the original FUN-LOV system (Figure 6A), confirming the importance of plasmid copy numbers and promoter strength in these optogenetic systems [9,17]. Similarly, the HAP-LOV switch showed an 18.8-fold increment in luciferase expression compared to FUN-LOV (Figure 6A), confirming the DBD as the protein module with the highest impact on the transcriptional response of optogenetic switches based on the Y2H system, such as FUN-LOV. The HAP-LOV switch variants also showed significant increments in luciferase expression compared to FUN-LOV (Figure 6A). These systems demonstrated 26-fold (HAP-LOV^p65^), 31.7-fold (HAP-LOV^p65-LS^), and 41.9-fold (HAP-LOV^LS^) increases in the luciferase levels compared to FUN-LOV, respectively (*t*-test, *p* < 0.01, Figure 6A). Interestingly, the multiple comparison statistical analysis among optogenetic systems (one-way ANOVA and Tukey’s multiple comparisons test), confirmed that all the assayed optogenetic systems, except FUN-LOV^VP16^ and FUN-LOV^p65^, showed statistically significant differences in the maximal luciferase expression compared to FUN-LOV (Supplementary Table S1). Altogether, these results confirm the observation that HAP-LOV^LS^ induces, among the tested systems, the strongest levels of activation by blue light, as measured by a luciferase reporter.

Finally, we calculated the fold change in luciferase expression (fold induction) reached by each optogenetic system upon blue-light stimulation compared to its background expression in constant darkness condition (Figure 6B). For this, the maximal raw luciferase expression measured during a blue-light pulse of 120 min was divided by the average raw luciferase expression measured in darkness prior to the blue-light pulse (Supplementary Table S2). Interestingly, the FUN-LOV^LS^ system showed an average 2870.7-fold induction (Figure 6B), which is higher than the fold-induction previously reported for FUN-LOV in the BY4741 genetic background (500-fold) or in the BY4741 *gal4∆*/*gal80∆* strain (1300-fold) [8]. Importantly, the higher fold induction observed in FUN-LOV^LS^ is the consequence not only of an increment in luciferase expression upon blue-light stimulation, but also of its low background in the darkness condition, a hallmark observed in all of the tested FUN-LOV variants (Supplementary Table S2). Furthermore, the background luciferase expression in constant darkness was approximately 22 times lower in the FUN-LOV variants compared to the HAP-LOV systems (Supplementary Table S2). Therefore, the HAP-LOV systems exhibited higher luciferase expression upon blue-light illumination but only a moderate increase in fold induction (Figure 6B). Finally, FUN-LOV^LS^ showed a statistically significant difference in the reporter fold-induction compared to all the assessed systems (Supplementary Table S3, one-way ANOVA, and Tukey’s multiple comparisons test). Altogether, our results confirm the importance of achieving low background expression in the darkness condition (measured here as destabilized luciferase levels) as a key factor impacting the dynamic range of yeast optogenetic systems.

As explained earlier, in the HAP-LOV switch and its variants, the Gal4p DBD of FUN-LOV is replaced by the Hap1p DBD. This modification changes the promoter specificity of the system, since Hap1p targets the *CYC1* promoter, which has a complex transcriptional regulation. Indeed, this promoter includes an upstream activation sequence (UAS1) for Hap1p binding [30], one UAS2 sequence for glucose repression through Mig1p binding [51], and one region for the CCAAT-binding complex, responsible for the regulation of respiratory genes [52]. Interestingly, the core *CYC1* promoter, a region between −1 and −250 bp approximatively (relative to the translational start site) that does not contain UASs, includes two TATA boxes binding the TFIID without the necessity of upstream regulatory factors [53]. Furthermore, the core *CYC1* promoter is depleted of nucleosomes, allowing the binding of TFIID and RNA polymerase II even in its repressed state [54]. This evidence suggests that the *CYC1* promoter is ready to be activated (i.e., poised), and that the recruitment of TFIID and RNA polymerase II are not limiting factors in its activation [54]. Therefore, we hypothesize that the higher luciferase expression obtained with HAP-LOV was a consequence of the complex regulatory layers co-occurring on the *CYC1* promoter region.

Importantly, during recent decades, Gal4-based systems have become a pivotal orthogonal transcriptional tool in several animal models [24,25,26,27]. For instance, they have been amply used to control gene expression (under the control of Gal4 UAS) in specific cell types by modulating where (and also when) Gal4 is expressed [24,25]. Likewise, several optogenetic switches have also taken advantage of Gal4 orthogonality and have chosen its DBD as part of their structure [9,55]. However, Gal4 DBD-based optogenetic systems cannot be easily combined with other expression circuits already containing Gal4-modules (Gal4/Gal80 cell-specific expression, etc.). Thus, the use of other Zn(II)_2_Cyc_6_ family members offers some advantages, as they also hold the potential to be utilized as orthogonal controllers in animal systems. Indeed, Hap1p recognizes sequences that are similar to Gal4, yet different enough to provide specific recognition of cognate promoters. Future studies will be focused on assessing the utility of this and other Zn(II)_2_Cyc_6_ DBD-based optogenetic switches in systems other than fungi.

## 3. Materials and Methods

### 3.1. Yeast Strains, Medium, and Culture Conditions

*S. cerevisiae* strain BY4741 (*MATa his3*Δ*1 leu2*Δ*0 met15*Δ*0 ura3*Δ*0*) was used as a background for gene deletions and transformations. The strains used and generated in this work were maintained in YDPA medium (2% glucose, 2% peptone, 1% yeast extract, and 2% agar) at 30 °C. Strains carrying plasmids with auxotrophic markers were maintained in synthetic complete (SC) medium (0.67% yeast nitrogen base without amino acids, 2% glucose, 0.2% dropout mix, and 2% agar) minus the corresponding amino acid (dropout mix). All the strains generated in this work and their genotypes are listed in Supplementary Table S4.

### 3.2. Illumination Conditions

We developed a custom illumination system for 96-well plates using LED RGB light panels (Kozdiko, Dongguan City, China). The LED RGB panels had 12 LED lights of 2W and were connected as an in-series circuit, generating a larger panel with a similar size to a 96-well plate (Supplementary Figure S9). The emission spectrum of the red, green, and blue lights in the LED RGB panels was determined using an HR2000 high-resolution spectrometer (Ocean Optics, Largo, FL, USA). The spectrum of the red, green, and blue lights corresponded to peaks at 647 nm, 523 nm, and 466 nm of light intensity, respectively (Supplementary Figure S7). All the experiments utilizing blue light (BL) were carried out using this custom illumination system, with 24 μmol m^−2^ s^−2^ of light intensity. Light intensity was measured using a LightScout quantum light meter (Spectrum Technologies Inc., Aurora, IL, USA).

### 3.3. Molecular Cloning and Strain Construction

The genetic constructs utilized in this work were assembled from PCR fragments using the in vivo yeast recombinational cloning method [56]. In the genetic construct derived from the FUN-LOV switch [8], the fragments were PCR-amplified from the plasmids encoding the FUN-LOV system (Supplementary Table S3). In addition, the DBD of the different TFs assayed were PCR-amplified from genomic DNA from the BY4741 strain. The VP16 and p65 ADs were PCR-amplified from previously described plasmids [55,57]. The overlapping PCR fragments were cloned into pRS423, pRS425, pRS313, or pRS315 plasmids by yeast recombinational cloning [56]. In the reporter genetic constructs, the promoter regions recognized by each TF were PCR-amplified from genomic DNA from the BY4741 strain. Importantly, we PCR-amplified wider promoter regions to avoid the exclusion of UAS or other regulatory elements (Table 1). The destabilized version of firefly luciferase [58] was PCR-amplified from our previously described reporter plasmids [8]. These overlapping PCR fragments were cloned into a pRS426 plasmid by yeast recombinational cloning [56]. All the PCR reactions were carried out using Phusion Flash high-fidelity PCR master mix (Thermo Scientific, USA). The PCR products were transformed into the BY4741 strain using the standard lithium acetate protocol [59]. The assembled plasmids were extracted from yeast using the Zymoprep Yeast Plasmid Miniprep Kit (Zymo Research, Irvine, CA, USA). Plasmids were then transformed in *E. coli* (DH5α strain) and its fragments were confirmed by standard colony PCR. Finally, the plasmids were confirmed by Sanger sequencing (Macrogen Inc., Seoul, Republic of Korea) and transformed into the BY4741 strain for optogenetic experiments (Supplementary Table S1). All the plasmids used and generated in this work are listed in the Supplementary Table S5.

We followed the one-step PCR deletion by recombination protocol to generate the *HAP1* or *CHA4* gene deletion (*hap1∆* or *cha4∆*) strains [60]. Briefly, the kanamycin (*KanMx*) antibiotic resistance cassette was amplified by PCR using Phusion Flash high-fidelity PCR master mix (Thermo Scientific, Waltham, MA, USA) and 70 bp primers containing regions for direct homologous recombination on the *HAP1* or *CHA4* loci. This enabled the swapping of the endogenous *HAP1* or *CHA4* ORFs in the BY4741 strain with the selection marker (Supplementary Table S4). All the primers used in this work are listed in Supplementary Table S6.

### 3.4. In Vivo Luciferase Expression

A destabilized version of the firefly luciferase was used as real-time reporter of gene expression in living yeast cells [58]. This luciferase version includes the ARE mRNA degradation sequence and a PEST protein degradation sequence [58], enabling the measurement of the transcriptional activity of this reporter gene under the control of the optogenetic system (measured as luciferase activity), as previously reported [8]. Luciferase expression was measured in vivo under different illumination conditions: constant darkness (DD), constant blue light (BL), and BL pulses of different lengths. These experiments were carried out using a Cytation 3 microplate reader (BioTek, Winooski, VT, USA), which allowed the measurement of both the optical density at 600 nm (OD_600 nm_) and the luminescence of the cell cultures over time. Briefly, yeast strains were grown overnight in a 96-well plate with 200 μL of SC medium at 30 °C under the DD condition. Then, 10 μL of these cultures was used to inoculate a new 96-well plate containing 190 μL (20-fold dilution) of fresh medium plus 1 mM luciferin. For DD and BL pulses treatments, OD_600 nm_ and luminescence readings were acquired every 10 min, running a continuous kinetic protocol with 30 s of shaking (285 cycles/min) before each measurement. For BL treatments, we used a discontinuous kinetic protocol, exposing the plate to the light source but keeping the aforementioned time measurement. All experiments were performed in six biological replicates.

### 3.5. Statistical Analysis

Statistical analyses performed in this work, including the *t*-test and one-way ANOVA with Tukey’s multiple comparisons test, were carried out using GraphPad Prism version 9.1.2.

## 4. Conclusions

Our results provide an experimental proof that an optogenetic switch based on an Y2H system, such as FUN-LOV, can readily be optimized to tune light-activated transcription of a target gene. Overall, the battery of optogenetic switches derived from FUN-LOV generated in this work enabled light-controlled transcription in yeast, yielding a broad range of expression levels. These optogenetic tools can be used in spectral multiplexing to combine different light inputs to control several target genes, including those involved in metabolic pathways or heterologous protein production. Furthermore, the HAP-LOV and FUN-LOV systems have the potential to be applied in mammalian cells or animal systems.

## Figures and Tables

**Table 1:** DNA-binding domains (DBDs) of transcription factors from the Zinc cluster family and their target promoter regions used in this work.

Transcription Factor	DBD Region (aa)	Target Promoter	Promoter Region Used (bp)
Hap1p [31]	38–187	*CYC1* [29]	−953 to −1
Dal81p [32]	124–273	*UGA1* [33]	−534 to −1
Thi2p	1–150	*THI6* [34]	−422 to −1
Ecm22p [35]	17–166	*ERG2* [36]	−492 to −1
Cat8p [37]	43–192	*ICL1* [38]	−830 to −1
Ppr1 [39,40]	1–150	*URA1* [41]	−1000 to −1
Lys14p [42]	133–282	*LYS9* [43]	−731 to −1
Aro80p [44]	1–150	*ARO9* [44]	−608 to −1
Cha4p [45]	18–167	*CHA1* [46]	−409 to −1
Gal4p (FUN-LOV) [8]	1–149	*GAL1* (FUN-LOV) [8]	−515 to −1

## Data Availability

The datasets supporting the reported results are available upon request from the corresponding author.

## Supplementary Materials

The following are available online at https://www.mdpi.com/article/10.3390/ijms22168538/s1. Figure S1: Raw luciferase expression in yeast strains carrying the FUN-LOV switch variants with different DNA-binding domains (DBDs). Figure S2: Growth curves for yeast strains carrying the FUN-LOV switch variants with different DNA-binding domains (DBDs). Figure S3: Light response of the Cha4p-LOV optogenetic switch in a cha4Δ yeast strain. Figure S4: Normalized luciferase expression in the FUN-LOV and HAP-LOV systems under blue-light pulses of different lengths. Figure S5: Raw data for the FUN-LOV and HAP-LOV systems under constant blue-light (BL) illumination. Figure S6: Normalized luciferase expression in the HAP-LOV and HAP-LOVp65 systems under blue-light pulses of different lengths. Figure S7: Molecular optimization of the promoter strength and plasmid copy number in the FUN-LOV switch. Figure S8: Molecular optimization of the promoter strength and plasmid copy number in the HAP-LOV switch. Figure S9: Illumination system used in our experiments. Table S1: Statistical comparison between optogenetic systems for the maximal nor-malized luciferase expression dataset. Table S2: Fold induction achieved by the optogenetic systems upon a blue-light (BL) pulse and measured as luciferase expression. Table S3: Statistical comparison between optogenetic systems for the luciferase fold-induction dataset. Table S4: Yeast strains used and generated in this work. Table S5: Plasmids used and generated in this work. Table S6: Primers used in this work.
