# Supplementary material for: Modular and Molecular Optimization of a LOV (Light–Oxygen–Voltage)-Based Optogenetic Switch in Yeast
Source: Int J Mol Sci. 2021 Aug 9;22(16):8538. doi: 10.3390/ijms22168538 (PMC8395189; doi:10.3390/ijms22168538)
Supplement: Supplementary file 1 [file ijms-22-08538-s001.zip › Supp_info_v2.pdf]

Supplementary Information

Modular and molecular optimization of a LOV (Light-Oxygen and Voltage) based optogenetic switch in yeast

Andres Romero[1,2,3], Vicente Rojas[1,2], Francisco Salinas[2,3], Luis F. Larrondo[1,2]*

1. Departamento de Genética Molecular y Microbiología, Facultad de Ciencias Biológicas, Pontificia Universidad Católica de Chile, Santiago, Chile.
2. ANID – Millennium Science Initiative – Millennium Institute for Integrative Biology (iBIO), Santiago, Chile.
3. Instituto de Bioquímica y Microbiología, Facultad de Ciencias, Universidad Austral de Chile, Valdivia, Chile.

*Corresponding author email: llarrondo@bio.puc.cl

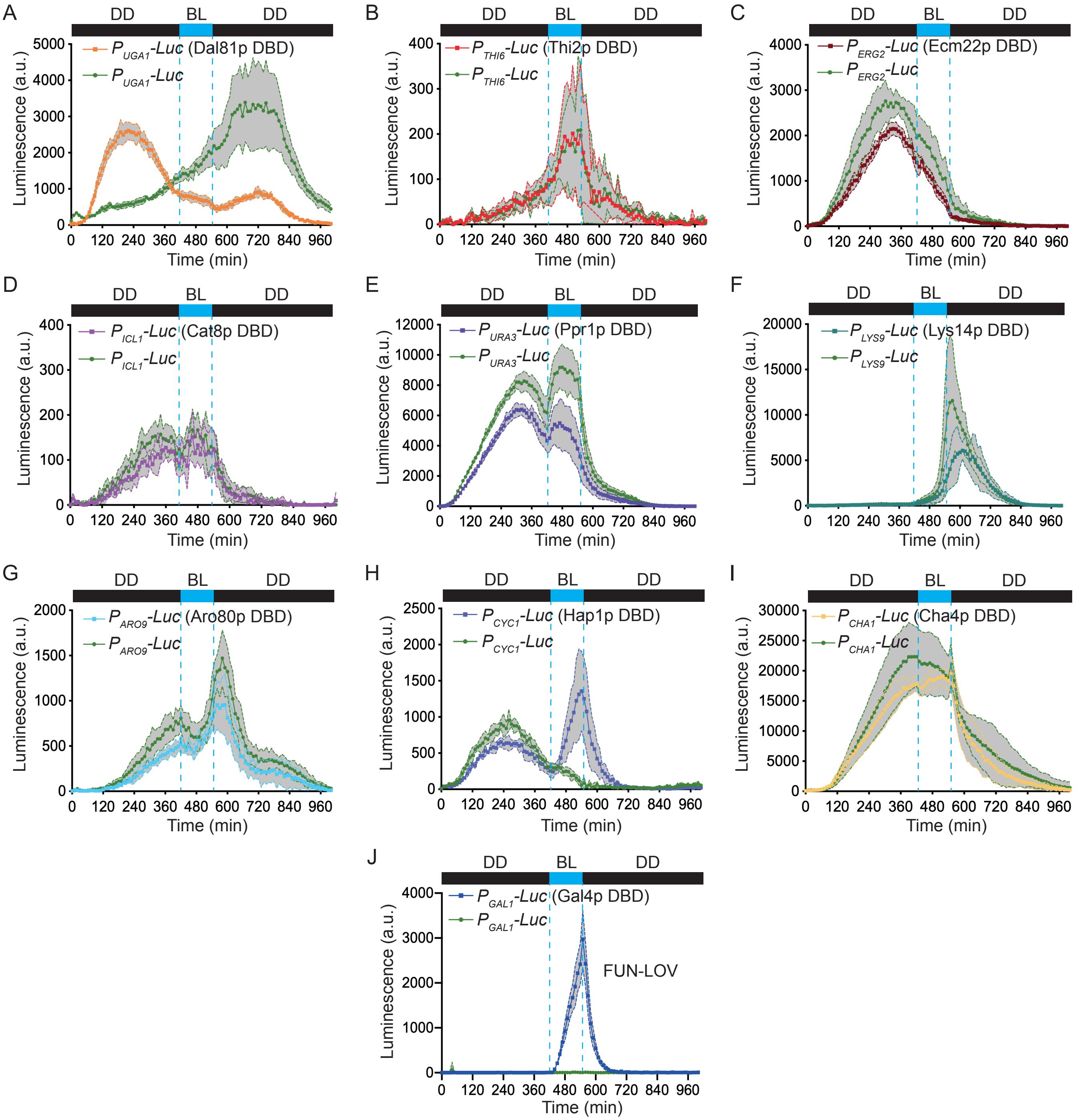

Supplementary Figure S1. Raw luciferase expression for the yeast strains carrying the FUN-LOV switch variants with different DNA-binding domains (DBD). (A) Dal80p DBD. (B) Thi2p DBD. (C) Ecm22p DBD. (D) Cat8p DBD. (E) Ppr1p DBD. (F) Lys14p DBD. (G) Aro9p DBD. (H) Hap1p DBD. (I) Cha4p DBD. (J) Gal4p DBD present in the FUN-LOV switch. In all the panels, the target promoter region recognized by each DBD is controlling the luciferase (*Luc*) expression and it was used as a control. The yeast strains were subjected to a blue-light (BL) pulse of 120 minutes indicated with blue dashed lines. The graphs show the average of six biological replicates with the standard deviation represented as shaded region. DD: constant darkness.

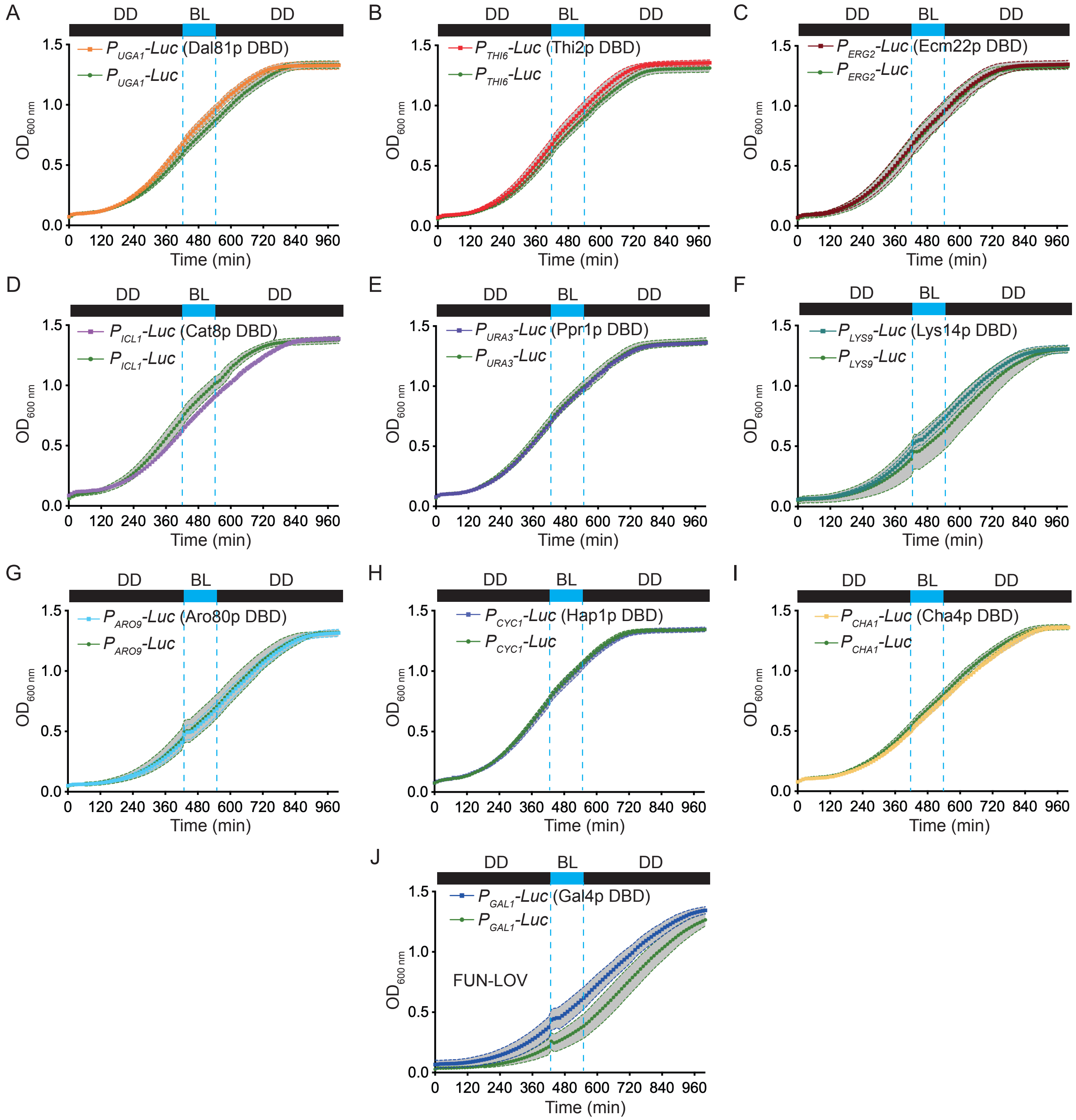

Supplementary Figure S2. Growth curves for the yeast strains carrying the FUN-LOV switch variants with different DNA-binding domains (DBD). (A) Dal80p DBD. (B) Thi2p DBD. (C) Ecm22p DBD. (D) Cat8p DBD. (E) Ppr1p DBD. (F) Lys14p DBD. (G) Aro9p DBD. (H) Hap1p DBD. (I) Cha4p DBD. (J) Gal4p DBD present in the FUN-LOV switch. The panels correspond to the same set of strains displayed in the Supplementary Figure S1. The yeast strains were subjected to a blue light (BL) pulse of 120 minutes indicated with blue dashed lines. The graphs show the average of six biological replicates with the standard deviation represented as shaded region. DD: constant darkness.

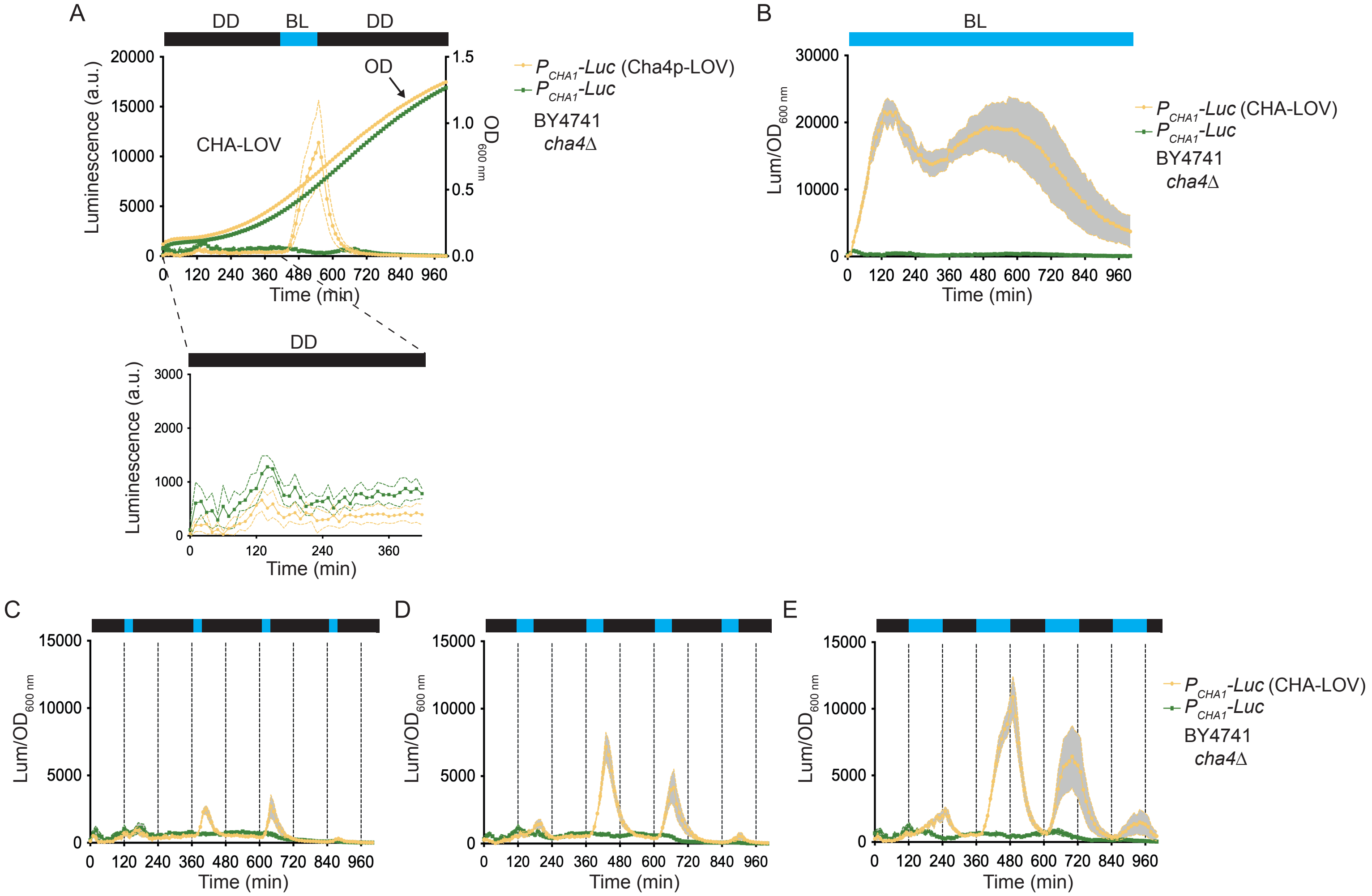

Supplementary Figure S3. Light response of the CHA4-LOV optogenetic switch in a *cha4Δ* yeast strain. (A) Raw luciferase expression activated by the CHA-LOV optogenetic switch upon a single 2 hours blue-light pulse. The OD (optical density) at 600 nm of each strain is shown on the right Y-axis. (B) Normalized luciferase expression for the CHA-LOV switch under constat blue-light condition. The yeast strain carrying the CHA-LOV switch was subjected to blue-light pulses of 30 minutes (C), 60 minutes (D), or 120 minutes (E) every 4 hours. The yeast strain carrying the *CHA1* (P_{CHA1}) promoter controlling the luciferase (*Luc*) expression was used as a control. In panel A, the average of raw luminescence and OD in six biological replicates is shown with the standard deviation (SD) represented as regions between dashed lines. The graphs in panels B, C, D, and E shows the average of normalized luciferase expression in six biological replicates with the SD represented as shaded region. Abbreviations: DD, constant darkness; BL, blue-light; a.u., arbitrary units of luminescence.

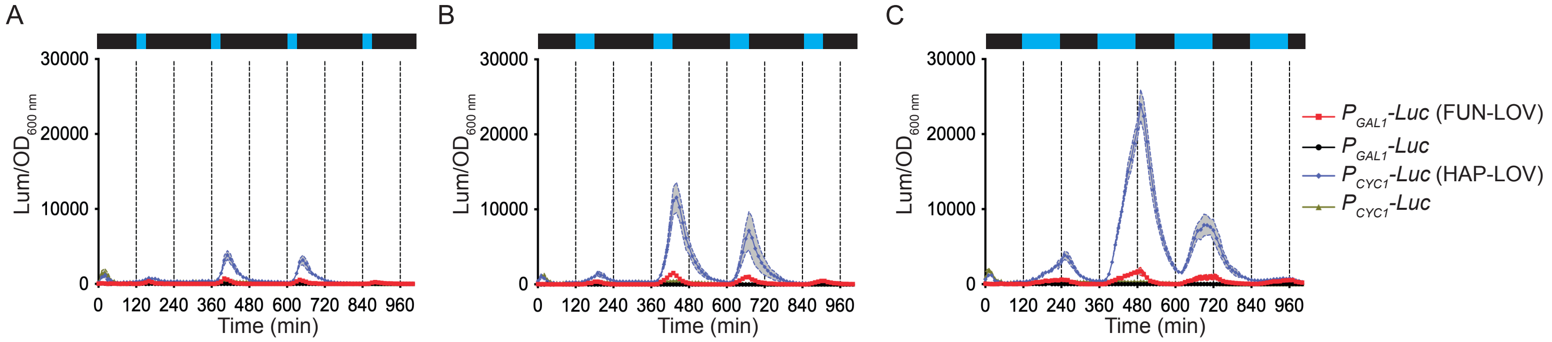

Supplementary Figure S4. Normalized luciferase expression activated by the FUN-LOV and HAP-LOV systems under blue-light pulses of different lengths. The yeast strains were subjected to blue-light pulses of 30 minutes (A), 60 minutes (B), or 120 minutes (C) every 4 hours. The yeast strains carrying the *GAL1* (P_{GAL1}) or *CYC1* (P_{CYC1}) promoters controlling the luciferase (*Luc*) expression were used as a control. The graphs show the average of normalized bioluminescence in six biological replicates with the standard deviation represented as shaded region.

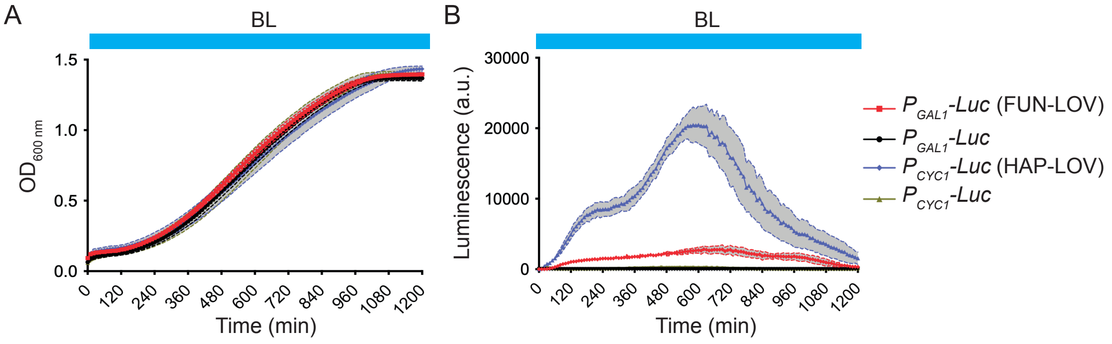

Supplementary Figure S5. Raw data for the FUN-LOV and HAP-LOV systems under constant blue-light (BL) illumination. Optical Density (OD) at 600 nm (A) and Luminescence (B). The yeast strains carrying the *GAL1* (P_{GAL1}) or *CYC1* (P_{CYC1}) promoters controlling the luciferase (*Luc*) expression were used as a control. The graphs show the average of six biological replicates with the standard deviation represented as shaded region. (a.u.): arbitrary units of luminescence.

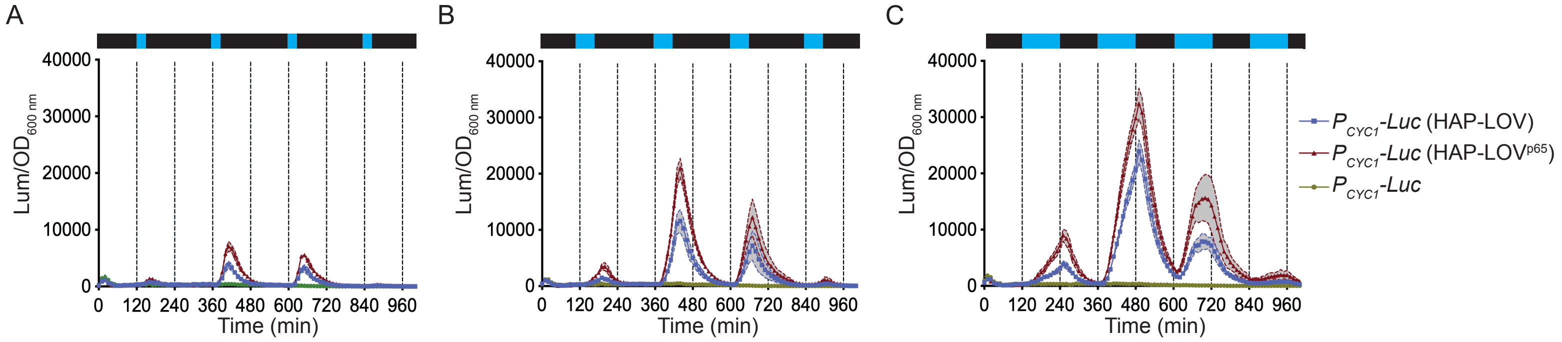

Supplementary Figure S6. Normalized luciferase expression activated by the HAP-LOV and HAP-LOV^{p65} systems under blue-light pulses of different lengths. The yeast strains were subjected to blue-light pulses of 30 minutes (A), 60 minutes (B), or 120 minutes (C) every 4 hours. The yeast strain carrying the *CYC1* (P_{CYC1}) promoter controlling the luciferase (*Luc*) expression was used as a control. The graphs show the average of normalized bioluminescence in six biological replicates with the standard deviation represented as shaded region.

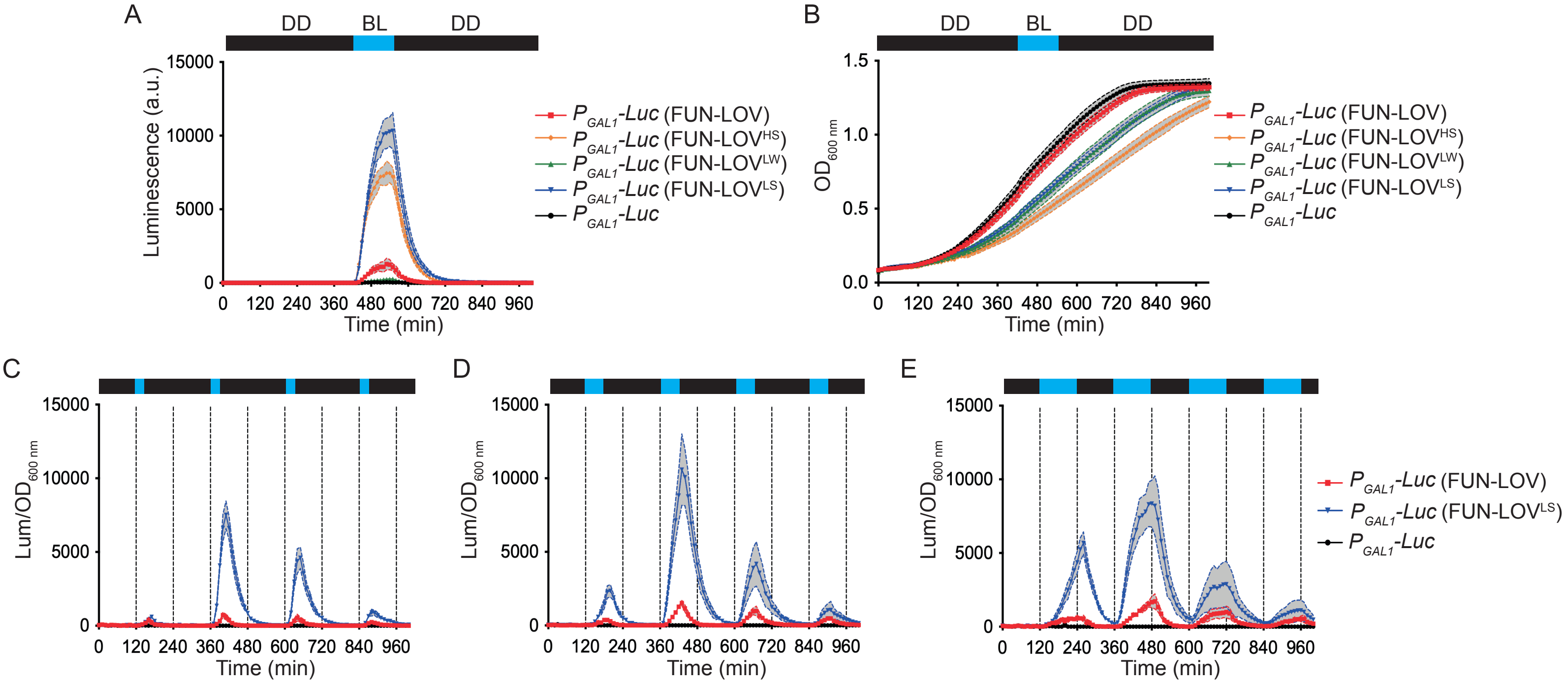

Supplementary Figure S7. Molecular optimization of the promoter strengh and plasmid copy number in the FUN-LOV switch. (A) Raw luciferase expression activated by different FUN-LOV variants. (B) Growth curves for different FUN-LOV variants. The yeast strains carrying the FUN-LOV and FUN-LOV^{LS} systems were subjected to blue-light pulses of different duration: 30 minutes (C), 60 minutes (D), or 120 minutes (E) every 4 hours. The yeast strain carrying the *GAL1* (P_{GAL1}) promoter controlling the luciferase (*Luc*) expression was used as a control. The graphs in panels C, D, and E shows the average of normalized luciferase expression in six biological replicates with the standard deviation represented as shaded region. Abbreviations: DD, constant darkness; BL, blue-light; H, high copy plasmid; L, low copy plasmid; S, strong promoter; W, weak promoter; a.u., arbitrary units of luminescence.

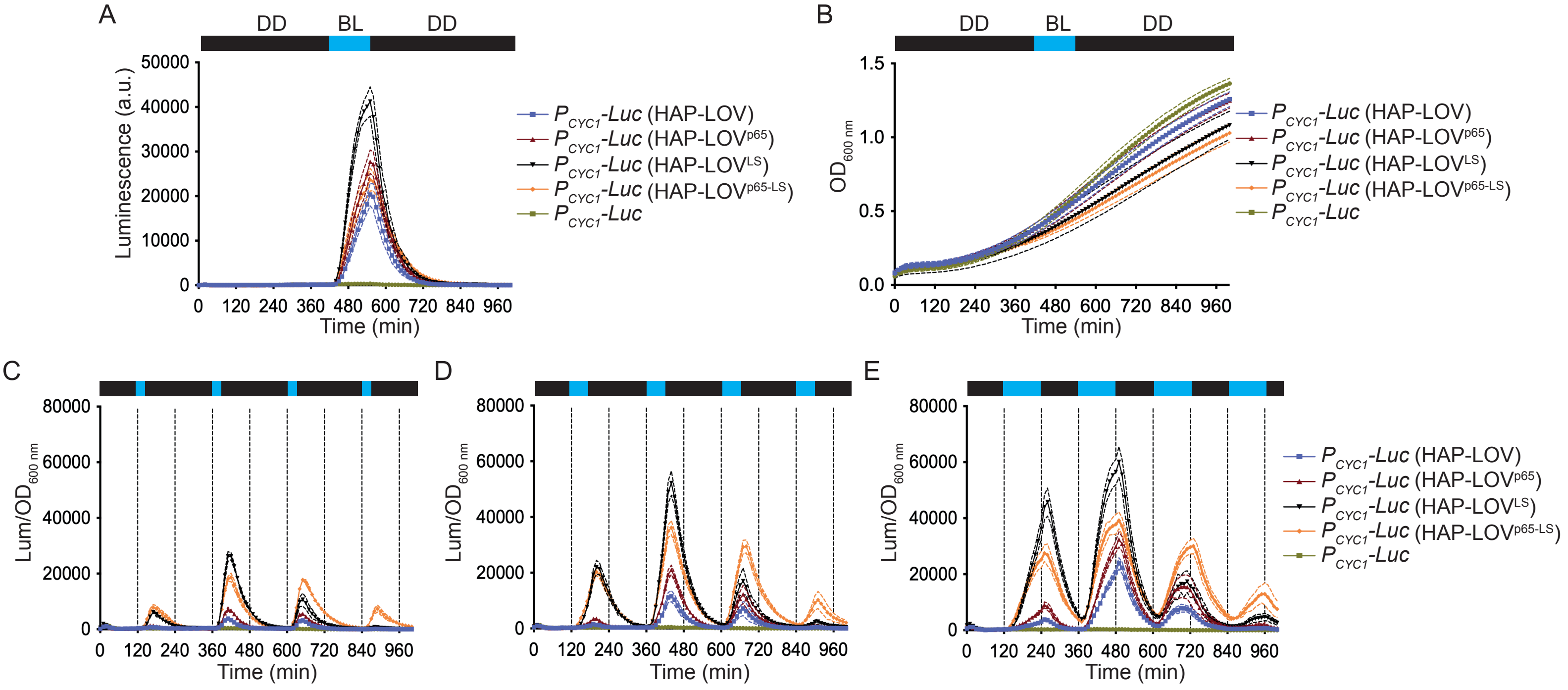

Supplementary Figure S8. Molecular optimization of the promoter strength and plasmid copy number in the HAP-LOV switch. (A) Raw luciferase expression activated by different HAP-LOV variants. (B) Growth curves for different HAP-LOV variants. The yeast strains carrying different version of the HAP-LOV switch were subjected to blue-light pulses of different duration: 30 minutes (C), 60 minutes (D), or 120 minutes (E) every 4 hours. The yeast strain carrying the *CYC1* (P_{CYC1}) promoter controlling the luciferase (*Luc*) expression was used as a control. The graphs in panels C, D, and E shows the average of normalized luciferase expression in six biological replicates with the standard deviation represented as shaded region. Abbreviations: DD, constant darkness; BL, blue-light; L, low copy plasmid; S, strong promoter; a.u., arbitrary units of luminescence.

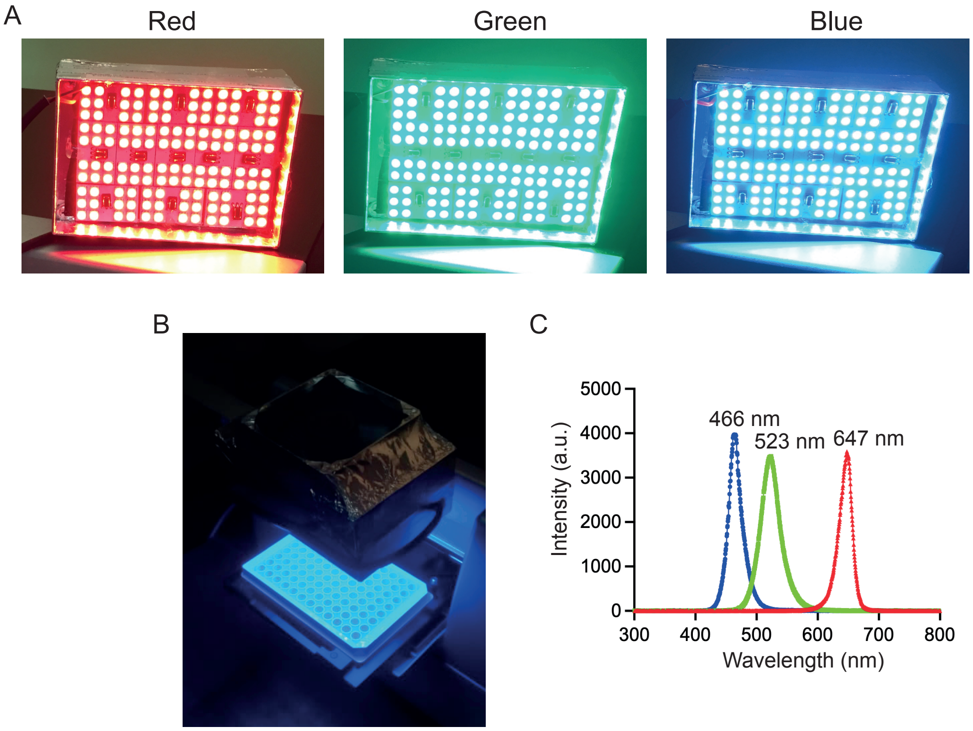

Supplementary Figure S9. Illumination system used in our experiments. (A) LED RGB panel developed in this work and enabling illumination with red, green, and blue lights. (B) 96-well plate under blue-light illumination using the LED panel. (C) Emission spectrum of the LED lights composing the panel.

Supplementary Table S1. Statistical comparison among optogenetic systems for the maximal normalized luciferase expression data set. One-way ANOVA and Turkey`s multiple comparisons tests were used in the statistical analysis.

Tukey's multiple comparisons test	Mean Diff.	95.00% CI of diff.	Below threshold?	Summary	Adjusted P Value
FUN-LOV vs. FUN-LOV^{VP16}	645.5	-5910 to 7201	No	ns	>0.9999
FUN-LOV vs. FUN-LOV^{p65}	37.18	-6518 to 6593	No	ns	>0.9999
FUN-LOV vs. FUN-LOV^{LS}	-14316	-20872 to -7760	Yes	****	<0.0001
FUN-LOV vs. HAP-LOV	-26870	-33426 to -20314	Yes	****	<0.0001
FUN-LOV vs. HAP-LOV^{VP16}	-18195	-24750 to -11639	Yes	****	<0.0001
FUN-LOV vs. HAP-LOV^{p65}	-37629	-44184 to -31073	Yes	****	<0.0001
FUN-LOV vs. HAP-LOV$^{p65\text{-}LS}$	-46333	-52888 to -39777	Yes	****	<0.0001
FUN-LOV vs. HAP-LOV^{LS}	-61686	-68242 to -55130	Yes	****	<0.0001
FUN-LOV^{VP16} vs. FUN-LOV^{p65}	-608.3	-7164 to 5947	No	ns	>0.9999
FUN-LOV^{VP16} vs. FUN-LOV^{LS}	-14961	-21517 to -8406	Yes	****	<0.0001
FUN-LOV^{VP16} vs. HAP-LOV	-27516	-34071 to -20960	Yes	****	<0.0001
FUN-LOV^{VP16} vs. HAP-LOV^{VP16}	-18840	-25396 to -12284	Yes	****	<0.0001
FUN-LOV^{VP16} vs. HAP-LOV^{p65}	-38274	-44830 to -31718	Yes	****	<0.0001
FUN-LOV^{VP16} vs. HAP-LOV$^{p65\text{-}LS}$	-46978	-53534 to -40423	Yes	****	<0.0001
FUN-LOV^{VP16} vs. HAP-LOV^{LS}	-62331	-68887 to -55776	Yes	****	<0.0001
FUN-LOV^{p65} vs. FUN-LOV^{LS}	-14353	-20909 to -7797	Yes	****	<0.0001
FUN-LOV^{p65} vs. HAP-LOV	-26907	-33463 to -20352	Yes	****	<0.0001
FUN-LOV^{p65} vs. HAP-LOV^{VP16}	-18232	-24787 to -11676	Yes	****	<0.0001
FUN-LOV^{p65} vs. HAP-LOV^{p65}	-37666	-44221 to -31110	Yes	****	<0.0001
FUN-LOV^{p65} vs. HAP-LOV$^{p65\text{-}LS}$	-46370	-52926 to -39814	Yes	****	<0.0001
FUN-LOV^{p65} vs. HAP-LOV^{LS}	-61723	-68279 to -55167	Yes	****	<0.0001
FUN-LOV^{LS} vs. HAP-LOV	-12554	-19110 to -5999	Yes	****	<0.0001
FUN-LOV^{LS} vs. HAP-LOV^{VP16}	-3879	-10434 to 2677	No	ns	0.5994
FUN-LOV^{LS} vs. HAP-LOV^{p65}	-23313	-29868 to -16757	Yes	****	<0.0001
FUN-LOV^{LS} vs. HAP-LOV$^{p65\text{-}LS}$	-32017	-38573 to -25461	Yes	****	<0.0001
FUN-LOV^{LS} vs. HAP-LOV^{LS}	-47370	-53926 to -40814	Yes	****	<0.0001
HAP-LOV vs. HAP-LOV^{VP16}	8675	2120 to 15231	Yes	**	0.0026
HAP-LOV vs. HAP-LOV^{p65}	-10759	-17314 to -4203	Yes	****	<0.0001
HAP-LOV vs. HAP-LOV$^{p65\text{-}LS}$	-19463	-26018 to -12907	Yes	****	<0.0001
HAP-LOV vs. HAP-LOV^{LS}	-34816	-41371 to -28260	Yes	****	<0.0001

HAP-LOV^{VP16} vs. HAP-LOV^{p65}	-19434	-25990 to -12878	Yes	****	<0.0001
HAP-LOV^{VP16} vs. HAP-LOV$^{p65\text{-}LS}$	-28138	-34694 to -21582	Yes	****	<0.0001
HAP-LOV^{VP16} vs. HAP-LOV^{LS}	-43491	-50047 to -36936	Yes	****	<0.0001
HAP-LOV^{p65} vs. HAP-LOV$^{p65\text{-}LS}$	-8704	-15260 to -2148	Yes	**	0.0025
HAP-LOV^{p65} vs. HAP-LOV^{LS}	-24057	-30613 to -17502	Yes	****	<0.0001
HAP-LOV$^{p65\text{-}LS}$ vs. HAP-LOV^{LS}	-15353	-21909 to -8797	Yes	****	<0.0001

ns: no significance.

Supplementary Table S2. Fold-induction achieved by the optogenetic systems upon a blue Blue-Light (BL) pulse and measured as luciferase expression.

System	Average raw luminescence in BL (a.u.)	Time of maximal luminescence (min)	Average raw luminescence in DD (a.u.)*	Fold induction (BL/DD)**
HAP-LOV^{LS}	41236.5 ± 3293.1	550	60 ± 44.2	686.7 ± 54.8
HAP-LOV^{P65-LS}	23696.3 ± 2554.1	550	35.4 ± 27.1	669.2 ± 72.1
HAP-LOV^{P65}	27724.7 ± 2627	550	54.9 ± 39.5	504.7 ± 47.8
HAP-LOV^{VP16}	16115.0 ± 1026.7	550	105 ± 58.6	153.5 ± 9.8
HAP-LOV	20366.8 ± 2556	550	66.2 ± 42.3	307.4 ± 38.6
FUN-LOV^{LS}	10326.7 ± 1246	550	3.6 ± 5.3	2870.7 ± 346.4
FUN-LOV^{P65}	1362.2 ± 492	540	2.6 ± 4.2	520.1 ± 187.9
FUN-LOV^{VP16}	824.5 ± 327.1	540	2.7 ± 4.3	301.1 ± 119.5
FUN-LOV	1282.5 ± 402.6	530	2.9 ± 4.5	438.7 ± 137.7

DD: constant darkness.
a.u.: arbitrary units of luminescence.
* Average raw luminescence measured in darkness condition previous to the blue-light pulse.
** One-way ANOVA and Tukey`s multiple comparisons test for this data set is shown in Supplementary Table S3.

Supplementary Table S3. Statistical comparison among optogenetic systems for the luciferase fold-induction data set. One-way ANOVA and Turkey`s multiple comparisons tests were used in the statistical analysis. The fold-induction was calculated for each optogenetic system dividing the maximal peak of luciferase expression upon a blue-light pulse by the average luciferase expression in darkness condition.

Tukey's multiple comparisons test	Mean Diff.	95.00% CI of diff.	Below threshold?	Summary	Adjusted P Value
FUN-LOV vs. FUN-LOV^{VP16}	137.6	-143.1 to 418.4	No	ns	0.8016
FUN-LOV vs. FUN-LOV^{p65}	-81.37	-362.1 to 199.4	No	ns	0.9889
FUN-LOV vs. FUN-LOV^{LS}	-2432	-2713 to -2151	Yes	****	<0.0001
FUN-LOV vs. HAP-LOV	131.3	-149.4 to 412.0	No	ns	0.8386
FUN-LOV vs. HAP-LOV^{VP16}	285.2	4.463 to 565.9	Yes	*	0.0439
FUN-LOV vs. HAP-LOV^{p65}	-65.99	-346.7 to 214.8	No	ns	0.9973
FUN-LOV vs. HAP-LOV^{p65-LS}	-230.5	-511.2 to 50.27	No	ns	0.1864
FUN-LOV vs. HAP-LOV^{LS}	-248.0	-528.7 to 32.75	No	ns	0.1219
FUN-LOV^{VP16} vs. FUN-LOV^{p65}	-219.0	-499.7 to 61.76	No	ns	0.2408
FUN-LOV^{VP16} vs. FUN-LOV^{LS}	-2570	-2850 to -2289	Yes	****	<0.0001
FUN-LOV^{VP16} vs. HAP-LOV	-6.308	-287.1 to 274.4	No	ns	>0.9999
FUN-LOV^{VP16} vs. HAP-LOV^{VP16}	147.6	-133.1 to 428.3	No	ns	0.7359
FUN-LOV^{VP16} vs. HAP-LOV^{p65}	-203.6	-484.3 to 77.14	No	ns	0.3290
FUN-LOV^{VP16} vs. HAP-LOV^{p65-LS}	-368.1	-648.8 to -87.34	Yes	**	0.0029
FUN-LOV^{VP16} vs. HAP-LOV^{LS}	-385.6	-666.4 to -104.9	Yes	**	0.0016
FUN-LOV^{p65} vs. FUN-LOV^{LS}	-2351	-2631 to -2070	Yes	****	<0.0001
FUN-LOV^{p65} vs. HAP-LOV	212.7	-68.07 to 493.4	No	ns	0.2749
FUN-LOV^{p65} vs. HAP-LOV^{VP16}	366.6	85.83 to 647.3	Yes	**	0.0031
FUN-LOV^{p65} vs. HAP-LOV^{p65}	15.38	-265.4 to 296.1	No	ns	>0.9999
FUN-LOV^{p65} vs. HAP-LOV^{p65-LS}	-149.1	-429.8 to 131.6	No	ns	0.7254
FUN-LOV^{p65} vs. HAP-LOV^{LS}	-166.6	-447.4 to 114.1	No	ns	0.5954
FUN-LOV^{LS} vs. HAP-LOV	2563	2283 to 2844	Yes	****	<0.0001
FUN-LOV^{LS} vs. HAP-LOV^{VP16}	2717	2436 to 2998	Yes	****	<0.0001
FUN-LOV^{LS} vs. HAP-LOV^{p65}	2366	2085 to 2647	Yes	****	<0.0001
FUN-LOV^{LS} vs. HAP-LOV^{p65-LS}	2202	1921 to 2482	Yes	****	<0.0001
FUN-LOV^{LS} vs. HAP-LOV^{LS}	2184	1903 to 2465	Yes	****	<0.0001
HAP-LOV vs. HAP-LOV^{VP16}	153.9	-126.8 to 434.6	No	ns	0.6910
HAP-LOV vs. HAP-LOV^{p65}	-197.3	-478.0 to 83.45	No	ns	0.3700

HAP-LOV vs. HAP-LOV$^{p65\text{-}LS}$	-361.8	-642.5 to -81.03	Yes	**	0.0037
HAP-LOV vs. HAP-LOV^{LS}	-379.3	-660.0 to -98.56	Yes	**	0.0020
HAP-LOV^{VP16} vs. HAP-LOV^{p65}	-351.2	-631.9 to -70.45	Yes	**	0.0053
HAP-LOV^{VP16} vs. HAP-LOV$^{p65\text{-}LS}$	-515.7	-796.4 to -234.9	Yes	****	<0.0001
HAP-LOV^{VP16} vs. HAP-LOV^{LS}	-533.2	-813.9 to -252.5	Yes	****	<0.0001
HAP-LOV^{p65} vs. HAP-LOV$^{p65\text{-}LS}$	-164.5	-445.2 to 116.3	No	ns	0.6118
HAP-LOV^{p65} vs. HAP-LOV^{LS}	-182.0	-462.8 to 98.73	No	ns	0.4786
HAP-LOV$^{p65\text{-}LS}$ vs. HAP-LOV^{LS}	-17.53	-298.3 to 263.2	No	ns	>0.9999

ns: no significance.

Supplementary Table S4. Yeast strains used and generated in this work.

Strain	Genotype*	Source
BY4741	*MATa his3Δ1 leu2Δ0 met15Δ0 ura3Δ0*	Euroscarf
hap1Δ	BY4741*; hap1Δ::KanMx*	This work
cha4Δ	BY4741*; cha4Δ::KanMx*	This work
P_{GAL1}-*Luc*	BY4741; plasmid 3	[8]
FUN-LOV	BY4741; plasmids 1, 2, and 3	[8]
FUN-LOV^{VP16}	BY4741; plasmids 1, 3, and 23	This work
FUN-LOV^{p65}	BY4741; plasmids 1, 3, and 22	This work
FUN-LOV^{HS}	BY4741; plasmids 3, 26, and 27	This work
FUN-LOV^{LS}	BY4741; plasmids 3, 28, and 29	This work
FUN-LOV^{LW}	BY4741; plasmids 3, 24, and 25	This work
P_{CYC1}-*Luc*	BY4741; plasmid 6	This work
P_{CYC1}-*Luc*	*hap1Δ*; plasmid 6	This work
P_{CHA1}-*Luc*	*cha4Δ*; plasmid 10	This work
CHA-LOV	*cha4Δ*; plasmids 2, 10, and 11	This work
HAP-LOV	*hap1Δ*; plasmids 2, 6, and 7	This work
HAP-LOV^{VP16}	*hap1Δ*; plasmids 6, 7, and 23	This work
HAP-LOV^{p65}	*hap1Δ*; plasmids 6, 7, and 22	This work
HAP-LOV^{LS}	*hap1Δ*; plasmids 6, 25, and 30	This work
HAP-LOV$^{p65\text{-}LS}$	*hap1Δ*; plasmids 6, 30, and 33	This work
P_{ARO9}-*Luc*	BY4741; plasmid 4	This work
Aro80p DBD	BY4741; plasmids 2, 4, and 5	This work
P_{LYS9}-*Luc*	BY4741; plasmid 8	This work
Lys14p DBD	BY4741; plasmids 2, 8, and 9	This work
P_{CHA1}-*Luc*	BY4741; plasmid 10	This work
Cha4p DBD	BY4741; plasmids 2, 10, and 11	This work
P_{THI6}-*Luc*	BY4741; plasmid 12	This work
Thi2p DBD	BY4741; plasmids 2, 12, and 13	This work
P_{ERG2}-*Luc*	BY4741; plasmid 12	This work
Ecm22p DBD	BY4741*;* plasmids 2, 14, and 15	This work
P_{ICL1}-*Luc*	BY4741*;* plasmid 16	This work
Cat8p DBD	BY4741*;* plasmids 2, 16, and 17	This work
P_{UGA1}-*Luc*	BY4741*;* plasmid 18	This work
Dal81p DBD	BY4741*;* plasmids 2, 18, and 19	This work
P_{URA3}-*Luc*	BY4741*;* plasmid 20	This work
Ppr1p DBD	BY4741*;* plasmids 2, 20, and 21	This work

* plasmid information in the Supplementary Table S5.

Supplementary Table S5. Plasmids used and generated in this work.

Plasmid number	Plasmid	Construct
1	pRS423-WC-1	P_{ADH1}-WC-1 *LOV-GAL4 DBD-ADH2*$_{ter}$
2	pRS425-VVD	P_{ADH1}-VVD-*GAL4 AD-ADH2*$_{ter}$
3	pRS426-P_{GAL1}-*Luc*	*KanMxRV*-P_{GAL1}-*Luc-CYC1*$_{ter}$
4	pRS426-P_{ARO9}-*Luc*	P_{ARO9}-*Luc-CYC1*$_{ter}$
5	pRS423-WC-1-*ARO80* DBD	P_{ADH1}-WC-1 LOV-*ARO80* DBD-*ADH2*$_{ter}$
6	pRS426-P_{CYC1}-*Luc*	P_{CYC1}-*Luc-CYC*$_{ter}$
7	pRS423-WC-1-*HAP1* DBD	P_{ADH1}-WC-1 LOV -*HAP1* DBD-*ADH2*$_{ter}$
8	pRS426-P_{LYS9}-*Luc*	P_{LYS9}-*Luc-CYC*$_{ter}$
9	pRS423-WC-1-*LYS14* DBD	P_{ADH1}-WC-1 LOV-*LYS14* DBD-*ADH2*$_{ter}$
10	pRS426-P_{CHA1}-*Luc*	P_{CHA1}-*Luc-CYC*$_{ter}$
11	pRS423-P_{ADH1}-WC-1-*CHA4* DBD	P_{ADH1}-WC-1 LOV-*CHA4* DBD-*ADH2*$_{ter}$
12	pRS426-P_{THI6}-*Luc*	P_{THI6}-*Luc-CYC*$_{ter}$
13	pRS423-WC-1-*THI2* DBD	P_{ADH1}-WC-1 LOV-*THI2* DBD-*ADH2*$_{ter}$
14	pRS426-P_{ERG2}-*Luc*	P_{ERG2}-*Luc-CYC1*$_{ter}$
15	pRS423-WC-1-*ECM22* DBD	P_{ADH1}-WC-1 LOV-*ECM22* DBD-*ADH2*$_{ter}$
16	pRS426-P_{ICL1}-*Luc*	P_{ICL1}-*Luc-CYC1*$_{ter}$
17	pRS423-WC-1-*CAT8* DBD	P_{ADH1}-WC-1 LOV-*CAT8* DBD-*ADH2*$_{ter}$
18	pRS426-P_{UGA1}-*Luc*	P_{UGA1}-*Luc-CYC1*$_{ter}$
19	pRS423-WC-1-*DAL81* DBD	P_{ADH1}-WC-1 LOV-*DAL81* DBD-*ADH2*$_{ter}$
20	pRS426-P_{URA3}-*Luc*	P_{URA3}-*Luc-CYC1*$_{ter}$
21	pRS423-WC-1-*PPR1* DBD	P_{ADH1}-WC-1 LOV-*PPR1* DBD-*ADH2*$_{ter}$
22	pRS425-VVD-*p65*	P_{ADH1}-VVD-SV40-P65-*ADHt2*$_{ter}$
23	pRS425-VVD-*VP16*	P_{ADH1}-VVD-SV40-VP16-*ADHt2*$_{ter}$
24	pRS313-WC-1 (W)	P_{ADH1}-WC-1 LOV-*GAL4* DBD-*ADH2*$_{ter}$
25	pRS315-VVD (W)	P_{ADH1}-VVD-*GAL4* AD-*ADH2*$_{ter}$
26	pRS423-WC-1 (S)	P_{TDH3}-WC-1 LOV-*GAL4* DBD-*ADH2*$_{ter}$
27	pRS425-VVD (S)	P_{TDH3}-VVD-*GAL4* AD-*ADH2*$_{ter}$
28	pRS313-WC-1 (S)	P_{TDH3}-WC-1 LOV-*GAL4* DBD-*ADH2*$_{ter}$
29	pRS315-VVD (S)	P_{TDH3}-VVD-*GAL4* AD-*ADH2*$_{ter}$
30	pRS313-WC-1-*HAP1* DBD (W)	P_{ADH1}-WC-1 LOV-*HAP1* DBD-*ADH2*$_{ter}$
31	pRS423-WC-1-*HAP1*-DBD (S)	P_{TDH3}-WC-1 LOV-*HAP1* DBD-*ADH2*$_{ter}$
32	pRS313-WC-1-*HAP1-DBD* (S)	P_{TDH3}-WC-1 LOV-*HAP1* DBD -*ADH2*$_{ter}$
33	pRS315-VVD-*p65* (S)	P_{TDH3}-VVD-*p65-ADH2*$_{ter}$

(W): weak promoter; (S): strong promoter

Supplementary Table S6. Primers used in this work.

Name	Orientation	Length (nt)	Sequence (5' - 3')	Description
oL3758	Fw	50	agcggataacaatttcacacaggaaacagcTAGGCGCATGCAACTTCTTT	Recombination of P_{ADH1} with pRS313, pRS315, pRS423 or pRS425
oL3148	Rv	50	GGTAACGCCAGGGTTTTCCCAGTCACGACGCCGGTAGAGGTGTGGTCAAT	Recombination of *ADH2*$_{ter}$ with pRS313, pRS315, pRS423 or pRS425
oL3084	Fw	50	AGCGGATAACAATTTCACACAGGAAACAGCATCGATGAATTCGAGCTCGT	Recombination of *KanMx* with pRS426
oL3070	Fw	50	CGACTCACTATAGGGAATATTAAGCTTACCATGGCCGATGCTAAGAACAT	Recombination of P_{GAL1} with *Luc*
oL2833	Rv	50	GCCCTTCTTAATGTTCTTAGCATCGGCCATGGTAAGCTTAATATTCCCTA	Recombination of P_{GAL1} with *Luc*
pL599	Fw	50	gtaacgccagggttttcccagtcacgacgtgggaagtcatagtaatagat	Recombination of pRS426 with P_{ARO9}
pL598	Fw	50	ataccacaattacactctctcatcgactcaatggccgatgctaagaacat	Recombination of P_{ARO9} with *LUC*
pL600	Rv	50	gcccttcttaatgttcttagcatcggccattgagtcgatgagagagtgta	Recombination of P_{ARO9} with *Luc*
pL597	Rv	50	gcggataacaatttcacacaggaaacagcttggatccttgcaaattaaag	Recombination of *CYC1*$_{ter}$ with pRS426
pL546	Fw	50	atgcaggtcaactatacccacagcggatccatgtctgctaagaaaaggcc	Recombination of *WC-1* with *ARO80 DBD*
pL545	Rv	50	gtttcccgaaggccttttcttagcagacatggatccgctgtgggtatagt	Recombination of *WC-1* with *ARO80 DBD*
pL548	Fw	50	ggaactgtagcaaaagaaggcgcaaaagaatttgtaatacgactcactat	Recombination of *ARO80 DBD ADH2*$_{ter}$
pL547	Rv	50	ggctcgccctatagtgagtcgtattacaaattcttttgcgccttcttttg	Recombination of *ARO80 DBD* with *ADH2*$_{ter}$
pL544	Rv	50	ggtaacgccagggttttcccagtcacgacggccggtagaggtgtggtcaa	Recombination of *ADH2*$_{ter}$ with *LacZ*
pL605	Fw	50	gtaacgccagggttttcccagtcacgacgtgttttagtgtgtgaatgaaa	Recombination of pRS426 with P_{CYC1}
pL604	Fw	50	acaaacacaaatacacacactaaattaataatggccgatgctaagaacat	Recombination of P_{CYC1} with *Luc*
pL606	Rv	50	gcccttcttaatgttcttagcatcggccattattaatttagtgtgtgtat	Recombination of P_{CYC1} with Luc
pL566	Fw	50	caggtcaactatacccacagcggatccatgagctctaactctccaccctt	Recombination of *WC-1* with *HAP1 DBD*
pL565	Rv	50	catgtgcaagggtggagagttagagctcatggatccgctgtgggtatagt	Recombination of *WC-1* with *HAP1 DBD*
pL568	Fw	50	caacaacagcaacagcagcaacaggaacaatttgtaatacgactcactat	Recombination of *HAP1 DBD ADH2*$_{ter}$
pL567	Rv	50	ggctcgccctatagtgagtcgtattacaaattgttcctgttgctgctgtt	Recombination of *HAP1 DBD ADH2*$_{ter}$
pL614	Fw	50	gtaacgccagggttttcccagtcacgacgtcccatgatattgtaaactaa	Recombination of pRS426 with P_{LYS9}
pL613	Fw	50	gagttatattaacgtattatatattttaatatggccgatgctaagaacat	Recombination of P_{LYS9} with *Luc*
pL615	Rv	50	gcccttcttaatgttcttagcatcggccatattaaaatatataatacgtt	Recombination of P_{LYS9} with *Luc*
pL570	Fw	50	caggtcaactatacccacagcggatccatgacccctaatcctgctgtaac	Recombination of *WC-1* with *LYS14 DBD*
pL569	Rv	50	agttgaagttacagcaggattaggggtcatggatccgctgtgggtatagt	Recombination of *WC-1* with *LYS14 DBD*
pL572	Fw	50	gaccttaccaccacaatgaatggatatgactttgtaatacgactcactat	Recombination of *LYS14 DBD ADH2*$_{ter}$
pL571	Rv	50	ggctcgccctatagtgagtcgtattacaaagtcatatccattcattgtgg	Recombination of *LYS14 DBD* with *ADH2*$_{ter}$
pL602	Fw	50	gtaacgccagggttttcccagtcacgacgttaatcgatgtgtccttgttt	Recombination of pRS426 with P_{CHA1}
pL601	Fw	50	agacaagagacaggaaaattaaccagcgagatggccgatgctaagaacat	Recombination of P_{CHA1} with *Luc*
pL603	Rv	50	gcccttcttaatgttcttagcatcggccatctcgctggttaattttcctg	Recombination of P_{CHA1} with *Luc*

pL554	Fw	50	caggtcaactatacccacagcggatccatgcttccgtccagtttaaagaa	Recombination of *WC-1* with *CHA4 DBD*
pL553	Rv	50	cacagatttctttaaactggacggaagcatggatccgctgtgggtatagt	Recombination of *WC-1* with *CHA4 DBD*
pL556	Fw	50	ttcaccaagaaaatgccttccgagagcccttttgtaatacgactcactat	Recombination of *CHA4 DBD* with $ADH2_{ter}$
pL555	Rv	50	ggctcgccctatagtgagtcgtattacaaaagggctctcggaaggcattt	Recombination of *CHA4 DBD* with $ADH2_{ter}$
pL620	Fw	50	gtaacgccagggttttcccagtcacgacgttattgcttataaaaactgat	Recombination of pRS426 with P_{THI6}
pL619	Fw	50	aagctaaattatttaaatcaaacagcggaaatggccgatgctaagaacat	Recombination of P_{THI6} with *Luc*
pL621	Rv	50	gcccttcttaatgttcttagcatcggccatttccgctgtttgatttaaat	Recombination of P_{THI6} with *Luc*
pL586	Fw	50	atgcaggtcaactatacccacagcggatccatggtcaatagtaagaggca	Recombination of *WC-1* with *THI2 DBD*
pL585	Rv	50	gcttctctgctgcctcttactattgaccatggatccgctgtgggtatagt	Recombination of *WC-1* with *THI2 DBD*
pL588	Fw	50	cggagattgaagatatacaataacgctgtgtttgtaatacgactcactat	Recombination of *THI2 DBD* with $ADH2_{ter}$
pL587	Rv	50	ggctcgccctatagtgagtcgtattacaaacacagcgttattgtatatct	Recombination of *THI2 DBD* with $ADH2_{ter}$
pL608	Fw	50	gtaacgccagggttttcccagtcacgacgtagcgttacgttcgataccttt	Recombination of pRS426 with P_{ERG2}
pL607	Fw	50	aaactaagactagcccagaccattatagccatggccgatgctaagaacat	Recombination of P_{ERG2} with *Luc*
pL609	Rv	50	tagcccagaccattatagccatggccgatgctaagaacattaagaagggc	Recombination of P_{ERG2} with *Luc*
pL562	Fw	50	caggtcaactatacccacagcggatccatggaactgattgaggttggggg	Recombination of *WC-1* with *ECM22 DBD*
pL561	Rv	50	tttcttgcccccaacctcaatcagttccatggatccgctgtgggtatagt	Recombination of *WC-1* with *ECM22 DBD*
pL564	Fw	50	tccaataatatggagaatcttttgttaccgtttgtaatacgactcactat	Recombination of *ECM22 DBD* with $ADH2_{ter}$
pL563	Rv	50	ggctcgccctatagtgagtcgtattacaaacggtaacaaaagattctcca	Recombination of *ECM22 DBD* with $ADH2_{ter}$
pL611	Fw	50	gtaacgccagggttttcccagtcacgacgtatatccatcacctacgtcgc	Recombination of pRS426 *with* P_{ICL1}
pL610	Fw	50	tagcataacataacaaaaagtcaacgaaaaatggccgatgctaagaacat	Recombination of P_{ICL1} with *Luc*
pL612	Rv	50	gcccttcttaatgttcttagcatcggccatttttcgttgactttttgtta	Recombination of P_{ICL1} with *Luc*
pL550	Fw	50	caggtcaactatacccacagcggatccatgtccaagaacgtgaaggaagc	Recombination of *WC-1* with *CAT8 DBD*
pL549	Rv	50	gatcattgcttccttcacgttcttggacatggatccgctgtgggtatagt	Recombination of *WC-1* with *CAT8 DBD*
pL552	Fw	50	acggttaataagcagctacaaaatggcaaatttgtaatacgactcactat	Recombination of *CAT8 DBD* with $ADH2_{ter}$
pL551	Rv	50	ggctcgccctatagtgagtcgtattacaaatttgccattttgtagctgct	Recombination of *CAT8 DBD* with $ADH2_{ter}$
pL623	Fw	50	gtaacgccagggttttcccagtcacgacgttattgctaacgttcgcgatg	Recombination of pRS426 with P_{UGA1}
pL622	Fw	50	aaccgtcaataagaaatataactaagaacaatggccgatgctaagaacat	Recombination of P_{UGA1} with *Luc*
pL625	Rv	50	gcccttcttaatgttcttagcatcggccattgttcttagttatatttctt	Recombination of P_{UGA1} with *Luc*
pL558	Fw	50	caggtcaactatacccacagcggatccatggcagatcataatggcagtaa	Recombination of *WC-1* with *DAL81 DBD*
pL587	Rv	50	gttgctattactgccattatgatctgccatggatccgctgtgggtatagt	Recombination of *WC-1* with *DAL81 DBD*
pL560	Fw	50	ctagataatgttgaccaaatacagctatcatttgtaatacgactcactat	Recombination of *DAL81 DBD* with $ADH2_{ter}$
pL559	Rv	50	ggctcgccctatagtgagtcgtattacaaatgatagctgtatttggtcaa	Recombination of *DAL81 DBD* with $ADH2_{ter}$
oL5101	Fw	72	cgttgtaaaacgacggccagtgagcgcgcgtttcaattcatcattttttttttattct tttttttgatttcg	Recombination of P_{URA1} with pRS426
oL5038	Rv	53	GCCCTTCTTAATGTTCTTAGCATCGGCCATgatttatcttc gtttcctgcagg	Recombination of P_{URA1} with *Luc*

oL1720	Fw	20	ATGGCCGATGCTAAGAACAT	Amplification of *Luc*
oL5037	Rv	54	gcaattaaccctcactaaagggaacaaaagctggtggatccttgcaaattaaag	Recombination of $CYC1_{ter}$ with pRS426
oL5031	Fw	53	ccatgcaggtcaactatacccacagcggatccATCTAGAACTGCATGTAAACG	Recombination of *WC-1* with *PPR1 DBD*
oL3599	Rv	21	gctgtgggtatagttgacctg	Amplification of *WC-1*
oL4261	Fw	22	TTTGTAATACGACTCACTATAG	Amplification of $ADH2_{ter}$
oL5032	Rv	50	GCTCGCCCTATAGTGAGTCGTATTACAAAGAAACTGAACTGTACTTTTTC	Recombination of *PPR1 DBD* with $ADH2_{ter}$
oL5269	Fw	50	CCCGAGCCTCCAAAAAAGAAGAGAAAGGTCcccacccaggctggggaagg	Recombination *VVD_SV40* with *p65 AD*
oL5268	Rv	20	ggcggtacccaattcGACCT	Amplification of *VVD_SV40*
oL4570	Fw	23	ATCTTTAATACGACTCACTATAG	Amplification of $ADH2_{ter}$
oL5270	Rv	50	GCTCGCCCTATAGTGAGTCGTATTAAAGATggagctgatctgactcagca	Recombination of *p65 AD* with $ADH2_{ter}$
oL5352	Fw	50	AAGAAGAGAAAGGTCgaattgggtaccgccgccccccgaccgatgtcag	Recombination *VVD_SV40* with *VP16 AD*
oL4569	Rv	50	GCTCGCCCTATAGTGAGTCGTATTAAAGATTTAcccaccgtactcgtcaattc	Recombination of *VP16 AD* with $ADH2_{ter}$
oL4568	Fw	50	ATGGGTTTCCAGTGCGAAACGGAAggatccgccccccgaccgatgtcag	Recombination *VVD* with *VP16 AD*
oL3150	Fw	50	agcggataacaatttcacacaggaaacagcATTTCAAAGAATACGTAAAT	Recombination P_{TDH3} with pRS423 or pRS425
oL5379	Fw	50	ATAAACAAACAAATATCTCATATAcatatgAAGAGCATTTACTCCAAAAG	Recombination of P_{TDH3} with *WC-1*
oL5378	Rv	50	AGTAAATGCTCTTcatatgTATATGAGATATTTGTTTGTTTATGTGTGTT	Recombination of P_{TDH3} with *WC-1*
oL5381	Fw	50	ATAAACAAACAAATATCTCATATAcatatgATGAGCCATACCGTGAACTC	Recombination of P_{TDH3} with *VVD*
oL5380	Rv	50	CGGTATGGCTCATcatatgTATATGAGATATTTGTTTGTTTATGTGTGTT	Recombination of P_{TDH3} with *VVD*
oL5519	Fw	70	GTAAGGAAATAGAAGAAAAAGAAAAAAAAAAAAAGGGAACAATAGGTTAGCGGGTTAATTAAGGCGCGCC	*hap1* deletion with *KanMX*
oL5520	Rv	70	TCCTATTACATTATCAATCCTTGCGTTTCAGCTTCCACTAATTTAGATGAATCGATGAATTCGAGCTCGT	*hap1* deletion with *KanMX*
oL5521	Fw	20	CTCAAGATACCGCAAGCACA	External primer to confirm *hap1* deletion
oL5522	Rv	20	GGCGCTACCATGAGAAATGT	External primer to confirm *hap1* deletion
oL5587	Fw	70	GTTTTCAAAAATAGCCCTTTTAAAACTCGAAGCTCTACACAATCGCAGCACgggttaattaaggcgcgcc	*cha4* deletion with *KanMX*
oL5588	Rv	70	acgagctcgaattcatcgatTCTTGAGTGAAGAGGATATGTTTACTTGAACAAATATTCTTTATGTAAT	*cha4* deletion with *KanMX*
oL5589	Fw	21	CAATATGGAAAACCACGCATA	External primer to confirm *cha4* deletion
oL5590	Rv	20	ATGAAAAGGACCCTAGGGCT	External primer to confirm *cha4* deletion
oL2090	Rv	21	ttcagaaacaactctggcgca	Internal primer for *KanMx*
oL2091	Fw	21	catcctatggaactgcctcgg	Internal primer for *KanMx*
